# Hepatic arterial infusion chemotherapy combined with lenvatinib and immune checkpoint inhibitor versus lenvatinib for advanced hepatocellular carcinoma: a multicenter study with propensity score and coarsened exact matching

**DOI:** 10.1007/s11547-025-01975-3

**Published:** 2025-03-12

**Authors:** Qunfang Zhou, Hui Li, Ye Liang, Ruixia Li, Xiaohui Wang, Wei Wang, Mingyu Liu, Feng Duan, Zhimei Huang

**Affiliations:** 1 Department of Interventional Radiology, 5th Medical Center of Chinese PLA General Hospital, 28 Fuxing Road, Beijing, 100853 China; 2 Department of Minimally Invasive Interventional Radiology, Sun Yat-sen University Cancer Center, Sun Yat-sen University, Dongfeng East Road 651, Guangzhou, 510260 Guangdong Province China; 3 Department of Liver Surgery, The First Affiliated Hospital of Sun Yat-sen University, 58 Zhongshan Er Road, Guangzhou, 510080 Guangdong Province China; 4 Department of Hepatobiliary Surgery, Hunan Provincial People’s Hospital, 61 Jiefang West Road, Changsha, Hunan Province China; 5 Department of General Surgery, The First Affiliated Hospital of Jinzhou Medical University, Renmin Road No. 2, Jinzhou, 121000 Liaoning Province China; 6 Department of Interventional Radiology, The Affiliated Shunde Hospital of Jinan University, Guizhou East Road 50, Foshan, Guangdong Province China

**Keywords:** Advanced hepatocellular carcinoma, Lenvatinib, Immune checkpoint inhibitor, Overall survival, Coarsened exact matching

## Abstract

**Purpose:**

Hepatic arterial infusion chemotherapy (HAIC) combined with lenvatinib (Len) and immune checkpoint inhibitor (ICI) in treating advanced hepatocellular carcinoma (HCC) still needs further confirmation. We aimed to evaluate the efficacy of HAIC combined with Len and ICI (HAIC + Len + ICI) versus Len alone in advanced HCC.

**Methods:**

A total of 290 patients in Len group and 349 patients in HAIC + Len + ICI group were analysed. Propensity score matching (PSM), inverse probability treatment weighting (IPTW), and coarsened exact matching (CEM) analyses were used to balance the bias between two groups. Mediation analysis of treatment type in survival was performed for analysis.

**Results:**

The median progression-free survival (PFS) was 5.9 ± 0.2 months in Len group and 9.2 ± 0.5 months in HAIC + Len + ICI group. The HAIC + Len + ICI group demonstrated significantly better PFS than the Len group across the entire cohort (hazard ratio [HR], 0.50; 95% CI 0.43–0.60; *P* < 0.001). This advantage in PFS was sustained in the PSM, IPTW, and CEM cohorts. HAIC + Len + ICI group also showed better overall survival (OS) than the Len group (HR, 0.38; 95% CI 0.31–0.46; *P* < 0.001). The OS was also superior in the PSM, IPTW, and CEM cohorts. The objective response rate (ORR) in HAIC + Len + ICI group was twice as high as that in Len group. Further mediation analysis showed tumor response at 3 and 6 months had different mediation effect on survival.

**Conclusions:**

HAIC combined with Len and ICI showed improved better OS and PFS than Len alone. This triple therapy could be considered as a first-line treatment for advanced HCC.

**Supplementary Information:**

The online version contains supplementary material available at 10.1007/s11547-025-01975-3.

## Introduction

Hepatocellular carcinoma (HCC) is the second leading cause of cancer-related deaths and the fourth most common cancer, with approximately 65% of patients in China diagnosed at an advanced stage [1]. Macrovascular invasion (MVI), primarily in the form of portal vein tumor thrombus (PVTT), is the most prevalent pattern, while the lungs and lymph nodes are also common sites of metastasis [2]. Advanced HCC usually has a poor prognosis, and the current strategy for managing advanced HCC is systemic therapy [3]. The National Comprehensive Cancer Network recommends atezolizumab plus bevacizumab as the preferred initial therapy for advanced HCC [4]. However, tyrosine kinase inhibitors (TKIs) or TKIs combined with immune checkpoint inhibitors (ICIs) are often recommended as system therapy in China [5].

Lenvatinib (Len) has been used as a therapeutic agent for advanced HCC since the REFLECT trial, but the effectiveness of monotherapy is limited [6]. ICI therapies, such as programmed death receptor 1 (PD-1) inhibitors, have garnered much attention from oncology researchers. Len combined with pembrolizumab has shown promising outcomes in clinical trials and is recommended [7]. However, the combination of Len and pembrolizumab, though it showed better clinical benefits than Len alone for advanced HCC in the LEAP-002 trial, did not meet the pre-specified significance for improved overall survival (OS) and progression-free survival (PFS) [8]. Locoregional therapy combined with Len and PD-1 inhibitor may provide new insights into the treatment of advanced HCC [9]. The LEAP-012 trial (NCT04246177) evaluated the safety and efficacy of Len and pembrolizumab combined with transarterial chemoembolization (TACE) versus TACE alone for advanced HCC. Thus, there is growing interest in combining locoregional and systemic treatments for advanced HCC [10].

Hepatic arterial infusion chemotherapy (HAIC) with oxaliplatin, 5-fluorouracil, and leucovorin (FOLFOX) has shown significant survival improvement and acceptable safety in advanced HCC [11]. Studies have demonstrated that HAIC can induce tumor antigen release and disrupt the immunosuppressive microenvironment, strongly enhancing the immune response and contributing to long-term antitumor immunity [12, 13]. Although studies have shown that HAIC combined with Len and ICI achieves superior survival benefits compared to Len in advanced HCC, further analysis is required, considering tumor characteristics such as primary versus recurrent HCC, and different subtypes of advanced HCC benefiting from the three regimens therapy. In this retrospective multicenter study, we comprehensively analyzed the efficacy and safety of HAIC combined with Len and ICI compared with Len alone for advanced HCC.

## Methods

### Patients

This study included patients diagnosed with advanced HCC between January 2018 and June 2023 across six hospitals. The follow-up period concluded on June 30, 2024. This multicenter analysis was conducted at the Chinese PLA General Hospital, Sun Yat-sen University Cancer Center, the First Affiliated Hospital of Sun Yat-sen University, the First Affiliated Hospital of Jinzhou Medical University, Hunan People’s Hospital, and the Second Affiliated Hospital of Guangzhou Medical University. The study was exempt from ethical committee approval due to its retrospective design.

The primary inclusion criteria were as follows: (1) clinical or pathological diagnosis of primary or recurrent HCC at Barcelona Clinic Liver Cancer (BCLC) stage C; (2) at least one measurable lesion based on Response Evaluation Criteria in Solid Tumors version 1.1 (RECIST v 1.1) [14]; (3) age 18 to 75 years; (4) HAIC combined with Len and ICI (HAIC + Len + ICI group) or Len alone (Len group) as first-line therapy; (5) PVTT type I, type II, and type III according to Cheng’s criteria [15] or hepatic vein tumor thrombus (HVTT); (6) Child–Pugh class A or B; (7) Eastern Cooperative Oncology Group performance status (ECOG) score of 0–2; (8) hemoglobin ≥ 8.5 g/dL, total bilirubin ≤ 30 mmol/L, ALT and AST ≤ 5 × upper limit of normal, serum creatinine ≤ 1.5 × upper limit of normal; (9) prothrombin time ≤ 18 s or international normalized ratio < 1.7; (10) ability to understand the protocol and agree to sign a written informed consent form.

The primary exclusion criteria were as follows: (1) recurrent or primary HCC at BCLC stage B; (2) lenvatinib as second therapy; (3) obstructive tumor thrombus involving the inferior mesenteric vein or vena cava; (4) serious medical comorbidities; (5) evidence of hepatic decompensation, including ascites, gastrointestinal bleeding, or hepatic encephalopathy; (6) history of organ allograft; (7) cardiac ventricular arrhythmias requiring anti-arrhythmic therapy; (8) evidence of bleeding diathesis; (9) incomplete data or lose follow-up within 3 months.

### Treatment

In the HAIC + Len + ICI group, patients underwent 2–6 cycles of HAIC, interval of each cycle was 3–4 weeks with the FOLFOX regimen. The HAIC procedure was performed by radiologists with more than 5 years of experience in interventional therapy for HCC. A microcatheter was placed into the proper hepatic artery according to the tumor's location. After the patient returned to the ward, the FOLFOX-based regimen was administered intra-arterially through a microcatheter. The regimen was administered via the hepatic artery as follows: 85 mg/m^2^ oxaliplatin from hour 0–2, 400 mg/m^2^ leucovorin from hour 2–4 on, 400 mg/m^2^ fluorouracil bolus at hour 5, and 2400 mg/m^2^ fluorouracil over 46 h. The repeated HAIC regimen was administered every 3 weeks, with patients receiving 2–6 cycles of HAIC. Patients were divided into two groups based on the number of HAIC cycles.

The prescribed dose of Len was 12 mg (body weight ≥ 60 kg) or 8 mg (body weight < 60 kg) orally once a day. The drug dose was reduced, or treatment was interrupted in patients who developed grade ≥ 3 severe adverse events (AEs) or any unacceptable grade 2 drug-related AEs. For ICI therapy (including sintilimab, toripalimab, tislelizumab, and camrelizumab), the dose was administered according to the drug instructions. Len or ICI was administered within 3–7 days of HAIC. Combination therapy was discontinued or adjusted for disease progression and unacceptable toxicity. In the event of grade ≥ 3 AEs, the continuation of Len was decided by the oncologist, and HAIC was discontinued in HAIC + Len + ICI group, and the continuation of Len and ICI was also decided by the oncologist. AEs during treatment were recorded according to the National Cancer Institute Common Terminology Criteria for Adverse Events 4.0 [16].

### Follow-up and outcomes

The follow-up period ended on June 30, 2024. Tumor response was evaluated using dynamic contrast-enhanced computed tomography (CT) or magnetic resonance imaging (MRI) every 6 weeks following RECIST v1.1 criteria, which classified responses as complete response (CR), partial response (PR), stable disease (SD), or progressive disease (PD) [14]. The primary endpoint was PFS. Secondary endpoints included OS, objective response rate (ORR), and disease control rate (DCR). PFS was defined as the time from the start of treatment to progressive disease based on independent radiologic review according to RECIST v 1.1 criteria or the final follow-up. OS was defined as the duration from the date of inclusion to death or the final follow-up. ORR was defined as the percentage of patients with a tumor response of CR and PR. DCR was defined as the percentage of patients with tumor response of CR, PR, and SD.

The progression patterns were classified into three types: type 1, progression in the liver only; type 2, progression to non-liver organs (such as bone, lymph nodes, and kidneys); and type 3, liver progression associated with other non-liver organs. PVTT was classified based on the extent of tumor thrombus extension in the portal venous system, according to Cheng’s criteria [15]: type I, tumor thrombus involving the segmental branches of the portal vein or higher; type II, tumor thrombus involving the right/left portal vein; and type III, tumor thrombus involving the main portal vein. Albumin-bilirubin (ALBI) grade was determined for each patient, based on a score calculated using the formula: ALBI score = (log10 bilirubin × 0.66) + (albumin × −0.085) [17]. MVI included PVTT and HVTT.

### Statistical analysis

Categorical variables were compared between groups using the Chi-square test and are presented as percentages. Continuous variables were presented as mean and standard deviation, and compared by the Kruskal–Wallis test. Survival curves for OS and PFS were constructed using the Kaplan–Meier method and compared using the log-rank test. Propensity score matching (PSM) analysis was used to balance the bias, matching patients 1:1 using the nearest neighbor method with a caliper of 0.05, as described in a previous study [18]. Variables with significance level of *P* < 0.100 in the univariate analyses were included in the multivariate analysis using the Cox regression model. To address potential bias from baseline characteristics, inverse probability of treatment weighting (IPTW) was conducted [19]. The balance of these characteristics was examined using standard mean difference (SMD). A SMD of less than 0.10 indicating adequate balance. For adjusted multivariate analysis, Cox proportional hazards regression was used to calculate the adjusted hazard ratios (HR) for PFS and OS between the two groups, with 95% confidence interval (CI) for the risk of events. A stratification analysis was performed to evaluate the two treatments at different variable levels.

A directed acyclic graph (DAG) was created to identify potential confounders. The selection of confounding factors and mediators primarily considered the following aspects: (1) the inherent characteristics of patients known before treatment; (2) variables with statistically significant difference univariable and multivariable Cox analysis as candidate variables; (3) reported confounders by previous literature; (4) experts’ consideration to be clinically significant. Causal mediation analysis was used to quantify the mediation effect of tumor response between treatment modality and survival. Sensitivity analyses were performed using coarsened exact matching (CEM) to identify approximately exact matches between patients in the two groups and minimize covariate imbalance and confounding bias. To account for immortal time bias, landmark analyses at 6 and 12 months were performed on the entire and PSM dataset.

All statistical tests were two-sided, and *P* < 0.05 was considered significant. Statistical analyses were performed using R software (version 4.1.3; http://www.r-project.org) and GraphPad Prism 8.0.2 (https://www.graphpad.com).

## Result

### Baseline characteristics

**Fig. 1:**
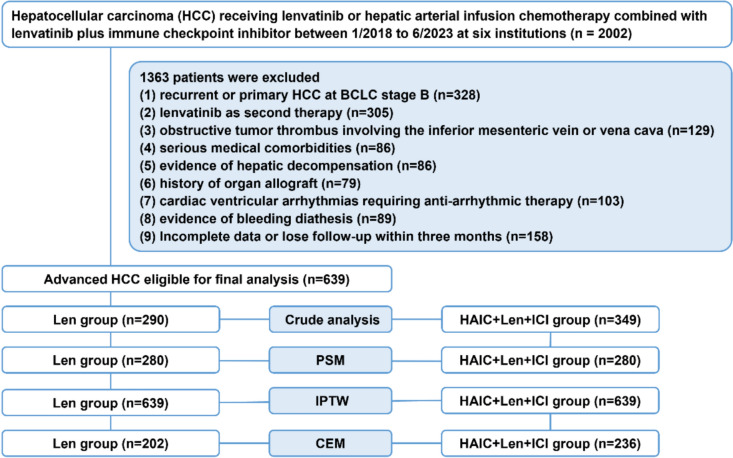
The flowchart of the study. HAIC + Len + ICI, hepatic arterial infusion chemotherapy combined with lenvatinib plus immune checkpoint inhibitor; HCC, hepatocellular carcinoma; IPTW, inverse probability of treatment weights; Len, lenvatinib; PSM, propensity score matching, CEM, coarsened exact matching

A total of 639 patients were included in this study according to the following inclusion criteria: 290 in the Len group and 349 in the HAIC + Len + ICI group. A flowchart of the patient selection process was shown in Fig. 1. Compared to the Len group, the HAIC + Len + ICI group had a higher proportion of patients with AFP ≥ 400 ng/mL (57.6% vs. 49.3%, *P* = 0.037) and ALBI grade 1 (39.5% vs. 29.7%, *P* < 0.001) in the entire cohort (Table 1). The variables were balanced after PSM, and no significant differences were observed between the two groups. Furthermore, IPTW and CEM were further applied to reduce bias. Table S1 presented the baseline characteristics of the IPTW and CEM. The SMD was well-balanced after IPTW (all < 0.1) (Figure S1).

**Table 1:** Baseline characteristics of entire cohort and PSM cohort

Characteristics	Entire cohort			PSM cohort		
	Len (*n* = 290)	HAIC + Len + ICI (*n* = 349)	*P* value	Len (*n* = 280)	HAIC + Len + ICI (*n* = 280)	*P* value
Sex			0.845			0.785
Male	259 (89.3%)	310 (88.8%)		249 (88.9%)	251 (89.6%)	
Female	31 (10.7%)	39 (11.2%)		31 (11.1%)	29 (10.4%)	
Age, year			0.834			0.912
≤ 60	240 (82.8%)	291 (83.4%)		231 (82.5%)	230 (82.1%)	
> 60	50 (17.2%)	58 (16.6%)		49 (17.5%)	50 (17.9%)	
Hepatitis			0.322			0.530
No	39 (13.4%)	38 (10.9%)		39 (13.9%)	34 (12.1%)	
Yes	251 (86.6%)	311 (89.1%)		241 (86.1%)	246 (87.9%)	
Anti-virus			0.267			0.435
No	74 (25.5%)	76 (21.8%)		74 (26.4%)	66 (23.6%)	
Yes	216 (74.5%)	273 (78.2%)		206 (73.6%)	214 (76.4%)	
Tumor number			0.165			0.926
≤ 3	84 (29.0%)	119 (34.1%)		83 (29.6%)	84 (30.0%)	
> 3	206 (71.0%)	230 (65.9%)		197 (70.4%)	196 (70.0%)	
Tumor size, cm			0.726			0.351
≤ 5	76 (26.2%)	82 (23.5%)		71 (25.4%)	57 (20.4%)	
> 5, ≤ 10	115 (39.7%)	142 (40.7%)		112 (40.0%)	123 (43.9%)	
> 10	99 (34.1%)	125 (35.8%)		97 (34.6%)	100 (35.7%)	
HCC type			0.705			0.342
Recurrent	82 (28.3%)	94 (26.9%)		81 (28.9%)	71 (25.4%)	
Primary	208 (71.7%)	255 (73.1%)				
Cirrhosis			0.688			0.865
No	130 (44.8%)	162 (46.4%)		129 (46.1%)	127 (45.4%)	
Yes	160 (55.2%)	187 (53.6%)		151 (53.9%)	153 (54.6%)	
Portal hypertension			0.446			1.000
No	204 (70.3%)	255 (73.1%)		200 (71.4%)	200 (71.4%)	
Yes	86 (29.7%)	94 (26.9%)		80 (28.6%)	80 (28.6%)	
ECGO PS			0.369			0.152
0	150 (51.7)	198 (56.7)		146 (52.1)	164 (58.6)	
1	108 (37.3)	121 (34.7)		103 (36.8)	95 (33.9)	
2	32 (11.0)	30 (8.6)		31 (11.7)	21 (7.5)	
MVI			0.885			0.929
No	94 (32.4%)	115 (33.0%)		94 (33.6%)	95 (33.9%)	
Yes	196 (67.6%)	234 (67.0%)		186 (66.4%)	185 (66.1%)	
Metastasis			0.494			0.861
No	107 (36.9%)	138 (39.5%)		104 (37.1%)	102 (36.4%)	
Yes	183 (63.1%)	211 (60.5%)		176 (62.9%)	178 (63.6%)	
HBV DNA			0.692			1.000
Negative	211 (72.8%)	249 (71.3%)		202 (72.1%)	202 (72.1%)	
Positive	79 (27.2%)	100 (28.7%)		78 (27.9%)	78 (27.9%)	
AFP, ng/mL			0.037			0.612
≤ 400	147 (50.7%)	148 (42.4%)		138 (49.3%)	132 (47.1%)	
> 400	143 (49.3%)	201 (57.6%)		142 (50.7%)	148 (52.9%)	
ALT, U/L			0.430			0.735
≤ 40	133 (45.9%)	171 (49.0%)		130 (46.4%)	134 (47.9%)	
> 40	157 (54.1%)	178 (51.0%)		150 (53.6%)	146 (52.1%)	
AST, U/L			0.503			0.786
≤ 40	90 (31.0%)	117 (33.5%)		88 (31.4%)	91 (32.5%)	
> 40	200 (69.0%)	232 (66.5%)		192 (68.6%)	189 (67.5%)	
ALBI grade			< 0.001			0.062
Grade 1	86 (29.7%)	138 (39.5%)		86 (30.7%)	92 (32.9%)	
Grade 2	176 (60.7%)	199 (57.0%)		168 (60.0%)	176 (62.9%)	
Grade 3	28 (9.7%)	12 (3.4%)		26 (9.3%)	12 (4.3%)	
Tumor size, cm	8.7 ± 4.4	8.8 ± 4.5	0.743	8.7 ± 4.4	9.0 ± 4.5	0.433
AFP, ng/mL	26541.9 ± 83974.9	31843.9 ± 109269.7	0.097	27143.0 ± 85251.2	32729.6 ± 120210.4	0.531
ALB, g/L	37.2 ± 4.3	38.0 ± 4.5	0.048	37.3 ± 4.3	37.5 ± 4.5	0.77
ALT, U/L	67.6 ± 117.1	64.2 ± 85.9	0.678	64.2 ± 98.0	66.9 ± 91.7	0.738
AST, U/L	107.7 ± 297.9	90.7 ± 114.9	0.33	107.6 ± 302.9	92.1 ± 120.1	0.429
Cre, mg/dL	71.5 ± 23.9	69.1 ± 22.9	0.210	71.5 ± 24.1	69.5 ± 24.6	0.360
LDH, U/L	300.9 ± 205.5	324.9 ± 268.1	0.267	301.9 ± 206.7	339.5 ± 293.8	0.119
TBIL, µmol/L	21.8 ± 24.8	18.5 ± 19.9	0.057	21.8 ± 25.2	19.4 ± 21.7	0.227
HGB, g/L	134.6 ± 68.8	134.4 ± 24.6	0.953	135.1 ± 69.9	132.9 ± 25.5	0.623
NEU, × 10^9^	4.8 ± 2.6	4.6 ± 3.0	0.432	4.8 ± 2.7	4.8 ± 3.1	0.931
PLT, × 10^9^	206.8 ± 153.5	190.0 ± 96.5	0.095	207.3 ± 154.0	194.4 ± 99.8	0.244

Abbreviations: AFP, alpha-fetoprotein; ALBI, albumin-bilirubin; ALT, alanine aminotransferase; AST, aspartate aminotransferase; ECOG, Eastern Cooperative Oncology Group performance status; HBV DNA, hepatitis B virus deoxyribonucleic acid; Len, Lenvatinib; MVI, macrovascular invasion

### PFS analysis between Len and HAIC + Len + ICI groups

**Fig. 2:**
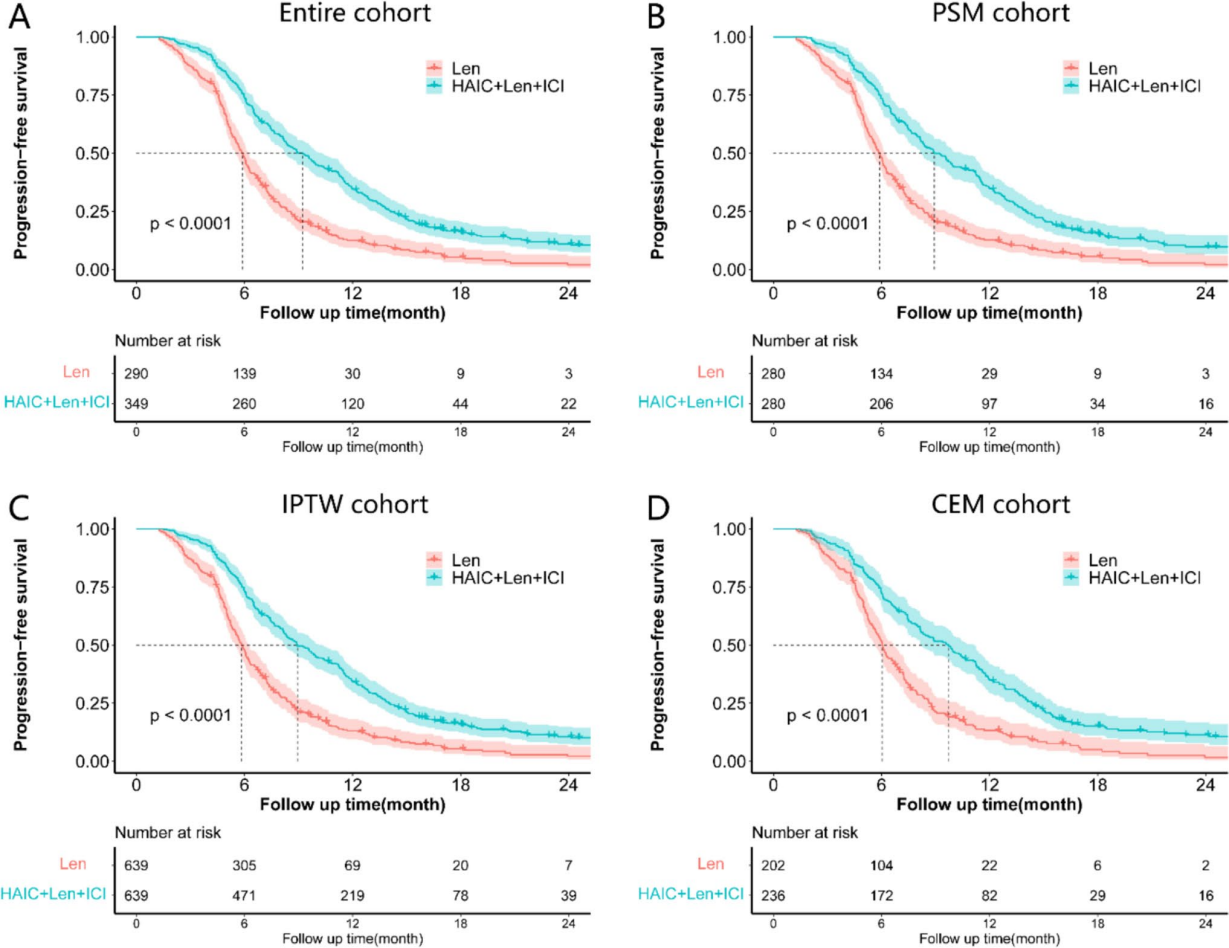
Survival curve of progression-free survival (PFS) in the entire cohort **A**, propensity score match (PSM) cohort **B**, inverse probability of treatment weighting (IPTW) cohort **C**, coarsened exact matching (CEM) cohort

The median follow-up time was 22.5 months (95% CI 6.0–43.0) for the Len group and 28.0 months (95% CI 6.0–52.5) for the HAIC + Len + ICI group. In the entire cohort, the median PFS was 5.9 ± 0.2 months (95% CI 5.6–6.2) for the Len group and 9.2 ± 0.5 months (95% CI 8.3–10.1) for the HAIC + Len + ICI group. In the PSM cohort, the median PFS was 5.9 ± 0.2 months (95% CI 5.6–6.2) for the Len group and 8.9 ± 0.5 months (95% CI 8.0–9.9) for the HAIC + Len + ICI group. The 6-, 12-, and 18-months PFS rates were 48.0%, 12.6%, and 2.0% for the Len group and 73.6%, 34.8%, and 15.4% for the HAIC + Len + ICI group, respectively (Table S2). Kaplan–Meier analysis for the entire cohort showed that the HAIC + Len + ICI group had better PFS than the Len group (HR, 0.50; 95% CI 0.43–0.60; *P* < 0.001) (Fig. 2A). This PFS advantage was sustained in the PSM (HR, 0.51; 95% CI 0.43–0.61; *P* < 0.001) (Fig. 2B), IPTW (HR, 0.52; 95% CI 0.43–0.62; *P* < 0.001) (Fig. 2C), and CEM (HR, 0.52; 95% CI 0.42–0.64; *P* < 0.001) (Fig. 2D) cohorts.

A multivariate Cox regression model showed that HAIC + Len + ICI treatment is an independent protective factor for PFS in the entire cohort (HR, 0.47; 95% CI 0.40–0.56; *P* < 0.001) (Table S3) and the PSM cohort (HR, 0.46; 95% CI 0.38–0.55; *P* < 0.001) (Table S4) after adjusting for demographic and tumor confounders. Stratification analysis of PFS demonstrated that HAIC + Len + ICI significantly outperformed Len except in female sex, hepatitis-negative status, and absence of MVI (Figure S2).

### OS analysis between Len and HAIC + Len + ICI groups

**Fig. 3:**
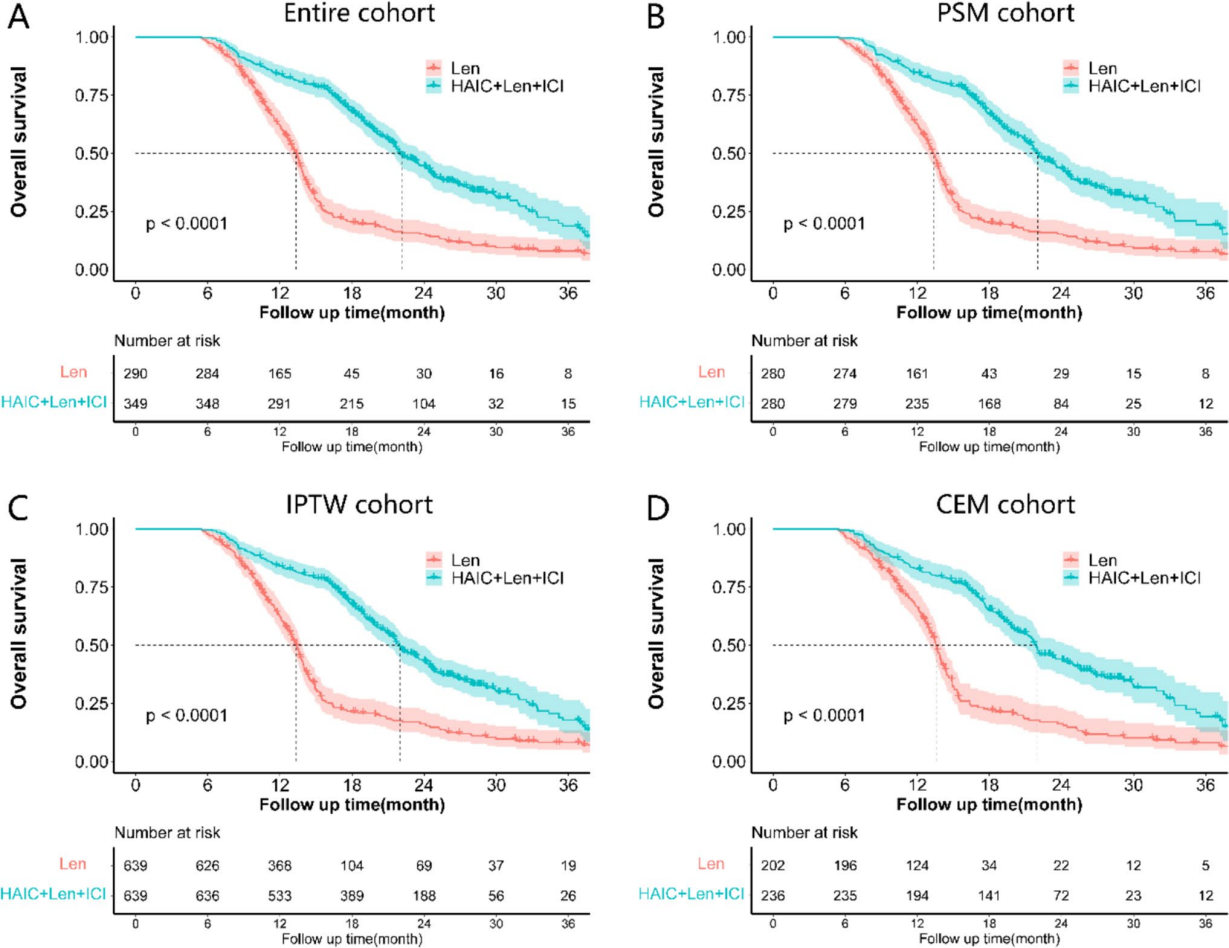
Survival curve of overall survival (OS) in the entire cohort **A**, propensity score match (PSM) cohort **B**, inverse probability of treatment weighting (IPTW) cohort **C**, coarsened exact matching (CEM) cohort **D**

At the data cut-off, 235 (81.0%) patients in the Len group and 213 (61.0%) patients in the HAIC + Len + ICI group had died of cancer. In the entire cohort, the median OS was 13.3 ± 0.2 months (95% CI 12.9–13.8) for the Len group and 22.0 ± 0.7 months (95% CI 20.7–23.3) for the HAIC + Len + ICI group. In the PSM cohort, the median OS was 13.3 ± 0.2 months (95% CI 12.8–13.8) for the Len group and 22.0 ± 0.8 months (95% CI 20.5–23.5) for the HAIC + Len + ICI group. The 1-, 2-, and 3-years OS rates were 62.3%, 15.3%, and 7.8% for the Len group and 84.6%, 43.9%, and 19.2% for the HAIC + Len + ICI group, respectively (Table S5). Kaplan–Meier analysis showed that the HAIC + Len + ICI group had better OS than the Len group (HR, 0.38; 95% CI 0.31–0.46; *P* < 0.001) (Fig. 3A). This OS advantage was consistent in the PSM (HR, 0.38; 95% CI 0.31–0.46; *P* < 0.001) (Fig. 3B), IPTW (HR, 0.39; 95% CI 0.32–0.48; *P* < 0.001) (Fig. 3C), and CEM (HR, 0.40; 95% CI 0.32–0.50; *P* < 0.001) (Fig. 3D) cohorts.

A multivariate Cox regression model showed that HAIC + Len + ICI treatment was an independent protective factor for OS in the entire cohort (HR, 0.33; 95% CI 0.27–0.41; *P* < 0.001) (Table S6) and the PSM cohort (HR, 0.34; 95% CI 0.27–0.42; *P* < 0.001) after adjusting for demographic and tumor confounders (Table S7). Stratification analysis of OS demonstrated that HAIC + Len + ICI significantly outperformed Len for all variables (Figure S3). Landmark analysis showed statistically significant improvements in OS with HAIC + Len + ICI for patients who lived longer than 6 months (HR, 0.40; 95% CI 0.33–0.49; *P* < 0.001) (Figure S4A) and 12 months (HR, 0.40; 95% CI 0.30–0.51; *P* < 0.001) (Figure S4B) in the entire cohort. Similar results were observed at 6 months (Figure S5A) and 12 months (Figure S5B) in the PSM cohort.

### Efficacy evaluation and progression analysis

Tumor response was assessed at 3 and 6 months according to RECIST v1.1 criteria. At the 6-months evaluation, the ORR and DCR were 19.7% (55/279) and 34.6% (97/279) in the Len group, and 51.4% (144/280) and 61.4% (172/280) in the HAIC + Len + ICI group respectively. The proportions of patients with CR, PR, SD, and PD differed significantly between the two groups in both the overall cohort (*P* < 0.001) and the PSM cohort (*P* < 0.001) (Table 2). Detailed values for the mediation analysis of tumor response on survival at 3 and 6 months were provided in Table S8. The mediation effect of tumor response revealed significant differences in PFS and OS between the Len and HAIC + Len + ICI groups in both the entire and PSM cohorts (*P* < 0.0001). The mediation effect and direct effect at 3 and 6 months were also different both in the entire and PSM cohort. The DAG was created to identify the confounders. The selection and classification of the confounders were informed by literature review and Cox analysis of PFS and OS (Figure S6).

**Table 2:** Efficacy outcomes in HAIC + Len + ICI and Len groups

Evaluation		Entire cohort			PSM cohort		
		Len (*n* = 290)	HAIC + Len + ICI (*n* = 349)	*P* value	Len (*n* = 280)	HAIC + Len + ICI (*n* = 280)	*P* value
3-months evaluation	CR	0	14 (4.0%)	< 0.001	0	13 (4.6%)	< 0.001
	PR	80 (27.6%)	189 (54.2%)		75 (26.8%)	150 (53.6%)	
	SD	148 (51.0%)	118 (33.8%)		145 (51.8%)	94 (33.6%)	
	PD	62 (21.4%)	28 (8.0%)		60 (21.4%)	23 (8.2%)	
	ORR	80 (27.6%)	203 (58.2%)	< 0.001	75 (26.8%)	163 (58.2%)	< 0.001
	DCR	228 (78.6%)	321 (92.0%)	< 0.001	220 (78.6%)	257 (91.8%)	< 0.001
6-months evaluation	CR	1 (0.3%)	18 (7.2%)	< 0.001	1 (0.4%)	16 (5.7%)	< 0.001
	PR	59 (20.4%)	165 (47.3%)		54 (19.3%)	128 (45.7%)	
	SD	42 (14.5%)	35 (10.0%)		42 (15.0%)	28 (10.0%)	
	PD	188 (64.8%)	131 (37.5%)		183 (65.4%)	108 (38.6%)	
	ORR	60 (20.7%)	183 (54.5%)	< 0.001	55 (19.7%)	144 (51.4%)	< 0.001
	DCR	102 (35.2%)	238 (62.5%)	< 0.001	97 (34.6%)	172 (61.4%)	< 0.001

The status of 3-month and 6-month evaluation of all tumors were assessed according to RECIST v 1.1 criteria

Abbreviations: CR, complete response; Len, Lenvatinib; PD, progressive disease; PR, partial response; SD, stable disease

Baseline comparisons between censored and non-censored cases of PFS were detailed for both the entire (Tables S9 and S10) and PSM cohorts (Tables S11 and S12), with no significant differences in patient characteristics post-PSM. Progression patterns between the Len and HAIC + Len + ICI groups were significant in both the entire (*P* = 0.019) and PSM (*P* = 0.028) cohorts (Table S13). Post-progression survival analysis indicated that the type 3 had the worst prognosis among the three types, while type 1 and type 2 showed no significant differences in either the overall (Figure S8A) or PSM cohorts (Figure S8B).

### Types of advanced HCC and prognosis

Advanced HCC was classified into three types: type I was characterized by localized advanced HCC with MVI only (including HVTT and PVTT); type II was defined as HCC with extrahepatic metastases only (including lungs, bones, and lymph nodes); and type III was defined as HCC with MVI and extrahepatic metastasis synchronously. Detailed information for advanced HCC in PSM cohort was presented in Table S14. Type III (with MVI and extrahepatic metastasis) was a poor predictor of PFS (Table S15) and OS (Table S16).

**Fig. 4:**
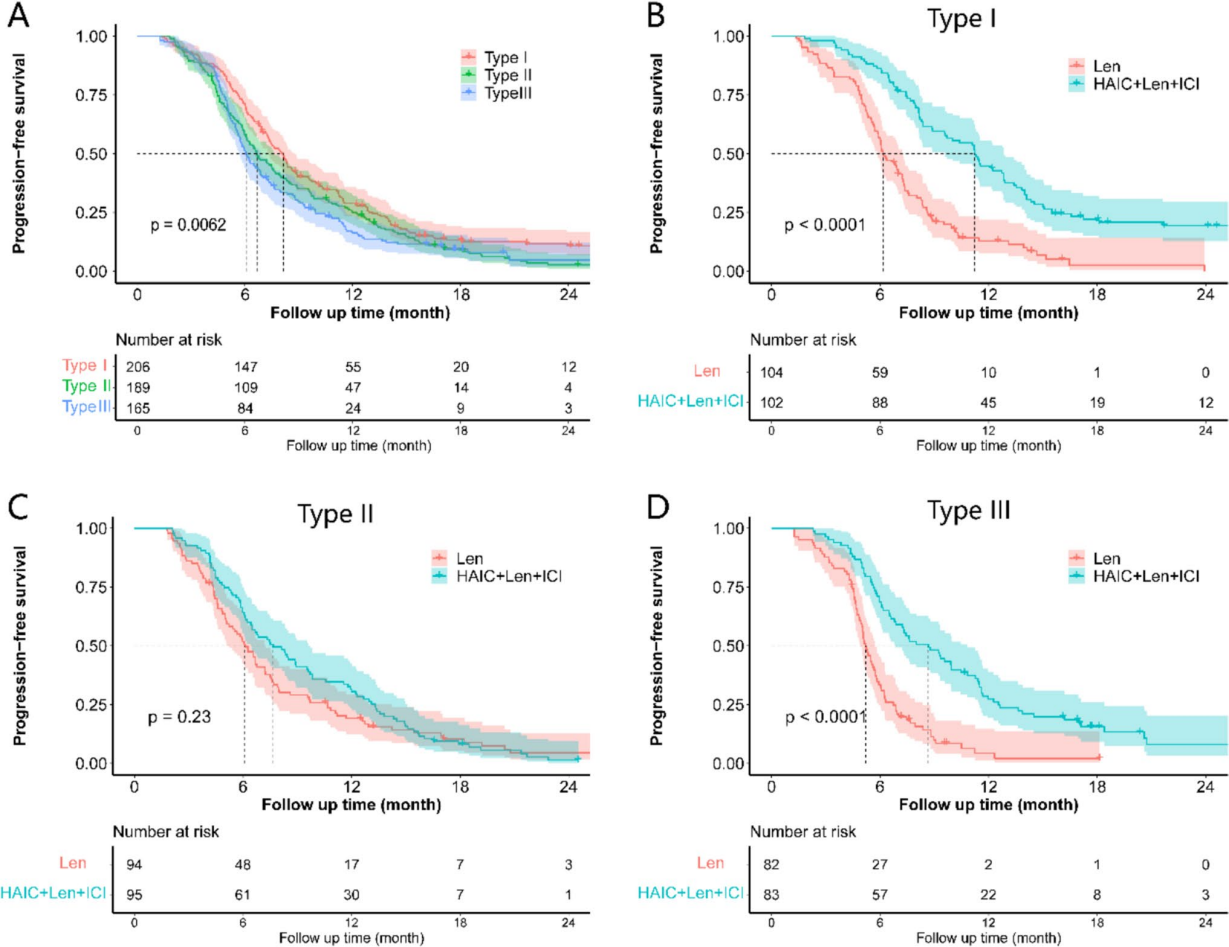
**A** Survival curve of progression-free survival (PFS) in the three types of advanced hepatocellular carcinoma. **B** PFS of type I between the Len and HAIC + Len + ICI groups. **C** PFS of type II between the Len and HAIC + Len + ICI groups. **D** PFS of type II between the Len and HAIC + Len + ICI groups. Type I was characterized by localized advanced HCC with macrovascular invasion (MVI) only; type II was defined as HCC with extrahepatic metastases only (including lungs, bones, and lymph nodes); and type III was defined as HCC with MVI and extrahepatic metastasis synchronously

Survival curves indicated that type III had shorter PFS compared to type I or type II (Fig. 4A). PFS was significantly better in the HAIC + Len + ICI group compared to the Len group for type I (Fig. 4B) and type III (Fig. 4D), but not for type II (Fig. 4C). Type III exhibited the shortest PFS among the three types (Figure S8A). In the HAIC + Len + ICI group, PFS was significantly longer than in the Len group for type I (Figure S8B), type II (Figure S8C), and type III (Figure S8D). Stratification analysis of advanced HCC, HVTT, PVTT, and metastatic organs on PFS (Figure S9) and OS (Figure S10) in the PSM cohort. The results showed no survival advantage for HAIC + Len + ICI over Len in type II of advanced HCC.

### Safety

Table S17 listed the most common AEs of all grades for each group. No treatment-related deaths were reported. Patients in the HAIC + Len + ICI group underwent 2–6 cycles of HAIC. The median duration of Len treatment was 11.0 months (range, 4.0–27.0) in the HAIC + Len + ICI group and 7.5 months (range, 4.0–23.0) in the Len group. The median duration of ICI treatment was 14.0 months (range, 4.0–36.0) in the HAIC + Len + ICI group. For Grade 3–4 AEs, Len or ICI administration was temporarily interrupted until the adverse effects were resolved.

## Discussion

In this multicenter analysis, we compared the prognoses of the HAIC + Len + ICI and Len alone groups. After adjusting for baseline characteristics through multi-matching analysis, the triple combination therapy (HAIC, Len, and ICI) demonstrated superior PFS and OS compared to Len alone. Although the increase in AE were observed with the combination therapy, there were deemed acceptable. These survival benefits were consistent across subgroup analyses. In our study, the median PFS and OS for HAIC + Len + ICI in advanced HCC were 8.9 months and 22.0 months, respectively, in the PSM cohort. Several studies have reported the effectiveness of HAIC in combination with systemic therapy for HCC [20]. Shi et al. reported that HAIC combined with Len and a PD-1 inhibitor showed better PFS compared to Len alone (11.1 months vs. 5.1 months) for advanced HCC [21]. Similarly, Guo et al. reported PFS and OS of 8.0 months and 27.0 months, respectively, for HAIC + Len + ICI in HCC with extrahepatic metastases [22]. The survival data in our study differ from those in these studies, which may be attributed to patient heterogeneity and the large-scale data from multiple centers.

This study has several strengths. First, the cohort of patients with advanced HCC was highly heterogeneous, including those with extrahepatic metastases, PVTT or HVTT, and multiple intrahepatic lesions. We conducted comprehensive comparisons in subgroup analyses to evaluate therapeutic differences. Second, to our knowledge, this is the largest study evaluating the efficacy and safety of combining HAIC with Len and ICI compared to Len alone as the first-line treatment for advanced HCC in a real-world setting. Third, we enhanced the reliability of the results by minimizing bias between the two groups using PSM, IPTW, ECM, and landmark analyses.

Standard treatments for advanced HCC include Atezolizumab plus Bevacizumab or Durvalumab plus Tremelimumab [23]. The median PFS and OS with Atezolizumab plus Bevacizumab are 19.2 months and 6.9 months, respectively, while for Durvalumab combined with Tremelimumab [24], they are 3.8 months and 16.4 months, respectively [25]. The OS and PFS in our study were longer than those reported for Atezolizumab plus Bevacizumab or Durvalumab plus Tremelimumab. Although standard systemic treatments are effective in prolonging OS, their impact on macrovascular invasion and extrahepatic metastasis remains unsatisfactory. Therefore, combining different therapies and leveraging their advantages is necessary for the treatment of advanced HCC.

There are several reasons to integrate HAIC with TKI and ICI for the treatment of advanced HCC. HAIC, a locoregional therapy, uses a catheter to directly administer anticancer drugs to tumors via the hepatic artery [26]. Oxaliplatin and fluorouracil in HAIC induce tumor cell death, which releases tumor antigens and proinflammatory cytokines [27], creating an immune-activated tumor environment that promotes dendritic cell and immune cell migration and maturation [28]. Len induces vascular normalization and enhances the efficacy of infiltrating immune cells [29]. ICIs activate cytotoxic T lymphocyte function, providing improved antitumor activity [30]. These mechanisms may explain the significant improvements in PFS and OS observed in our study.

The EMERALD-1 study (NCT03778957) found that TACE plus durvalumab and bevacizumab resulted in a median PFS of 15.0 months compared to 8.2 months with TACE alone (HR, 0.77; 95% CI 0.61–0.98; *P* = 0.032), while the LEAP-012 trial (NCT04246177) is ongoing and highly anticipated. Our study found that high intrahepatic or extrahepatic tumor burden was associated with poorer PFS and OS. HAIC, as an effective local therapy, can reduce intrahepatic tumor burden, potentially enhancing the efficacy of Len and ICIs as the tumor burden diminishes. However, it is essential to consider whether AEs might have a cumulative effect in combination therapy. In our study, the overall rate of AEs was higher in the HAIC + Len + ICI group than in the Len group. Patients in the HAIC + Len + ICI group experienced increased abdominal pain, nausea, fever, and temporary elevations in liver enzymes and hyperbilirubinemia, likely due to HAIC.

Tumor response by RECIST v 1.1 criteria was used as an indicator reflecting the treatment efficacy. The good tumor response including PR and CR usually presented the sign of better PFS and OS [31]. RECIST v 1.1 criteria was the standard of care for evaluating tumor response to systemic therapy in a quantitative and presumably objective manner [32]. Tumor response to systemic therapy especially for TKI or TKI combined ICI needed time before having obvious effectiveness. If imaging was conducted at 1-month for PR, and at 3-months met the criteria for SD, and at 6-months met the criteria for PD, then the best overall response was PR not SD as the subject. In most literature, only one non-specified time-point evaluation was used, while there was not comprehensively evaluated the therapeutic efficacy [8, 33]. In this study, we used two time-point evaluation (3 and 6 months) according to the RECIST v 1.1. The tumor response at 3 and 6 months had some difference, which could more accurate present the tumor response to different treatment. Mediation analysis was applied and the result revealed that different mediation effect in PFS and OS between HAIC + Len + ICI and Len groups was observed through the effect of tumor response. The mediation analysis was also revealed that the mediation effect and direct effect at 3 and 6 months were different. Thus, specified time-point evaluation might be more appropriate.

A key limitation of this study is its retrospective nature, which may introduce selection bias among patients receiving the two treatments. Although we employed multiple matching methods (PSM, IPTW, and ECM) to minimize between-group differences, endogenous differences may still exist. Therefore, further prospective randomized controlled trials are needed to validate our findings and provide stronger clinical evidence. Another limitation is that treatment decisions are influenced not only by expert recommendations but also by patients' medical profiles, financial considerations, and personal preferences. Additionally, the use of various ICIs, approved by the National Medical Products Administration and available in China, means that patients in the HAIC + Len + ICI group chose their ICI based on either the doctor’s recommendation or their own choice.

## Conclusion

In summary, the combination of HAIC with Len and an ICI regimen demonstrates enhanced efficacy, with significantly improved OS, PFS, and ORR, and manageable AE for advanced HCC. This triple therapy shows promising potential and can be considered as a first-line treatment for advanced HCC.

## Supplementary Information

Below is the link to the electronic supplementary material.

Supplementary file1 (DOCX 1689 KB)

## Data Availability

The data generated and analyzed in this study are included in the manuscript and supplementary material. The research data are not publicly available on ethical grounds. However, inquiries regarding all data analyzed in this study can be directed to the corresponding author.

## Abbreviations

ALT: Alanine aminotransferase
AST: Aspartate aminotransferase
AFP: Alpha-fetoprotein
ALBI: Albumin-bilirubin
CEM: Coarsened exact matching
CI: Confidence interval
CT: Computed tomography
CR: Complete response
DCR: Disease control rate
MVI: Macrovascular invasion
HAIC: Hepatic arterial infusion chemotherapy
HCC: Hepatocellular carcinoma
HR: Hazard ratio
HVTT: Hepatic vein tumor thrombus
ICI: Immune checkpoint inhibitor
IPTW: Inverse probability of treatment weighting
Len: Lenvatinib
MRI: Magnetic resonance imaging
ORR: Objective response rate
OS: Overall survival
PD: Progressive disease
PFS: Progression-free survival
PSM: Propensity score-matching
PR: Partial response
PVTT: Portal vein tumor thrombus
SD: Stable disease
SMD: Standardized mean difference
TACE: Transcatheter arterial chemoembolization
