# Supplementary material for: Hepatic arterial infusion chemotherapy combined with lenvatinib and immune checkpoint inhibitor versus lenvatinib for advanced hepatocellular carcinoma: a multicenter study with propensity score and coarsened exact matching
Source: Radiol Med. 2025 Mar 12;130(5):662–73. doi: 10.1007/s11547-025-01975-3 (PMC12106563; doi:10.1007/s11547-025-01975-3)
Supplement: Supplementary file 1 — Supplementary file1 (DOCX 1689 KB) [file 11547_2025_1975_MOESM1_ESM.docx]

**Hepatic arterial infusion chemotherapy combined with lenvatinib and immune checkpoint inhibitor versus lenvatinib for advanced hepatocellular carcinoma: A multicenter study with propensity score and coarsened exact matching**

**Table Legends**

**Table S1.** Baseline characteristics of IPTW cohort and ECM cohort.

**Table S2.** The median survival, 6-month, 12-month and 24-month of PFS between two groups.

**Table S3.** Univariable and multivariable analysis of prognostic factors for PFS in entire cohort.

**Table S4.** Univariable and multivariable analysis of prognostic factors for PFS in PSM cohort.

**Table S5.** The median survival, 1-years, 2-year and 3-year of OS between two groups.

**Table S6.** Univariable and multivariable analysis of prognostic factors for OS in entire cohort.

**Table S7.** Univariable and multivariable analysis of prognostic factors for OS in PSM cohort.

**Table S8.** Results of mediation analysis on the mediation effect of three-month and six-month evaluation in PFS and OS.

**Table S9.** Baseline characteristics of censored patients between HAIC+Len+ICI and Len groups in the entire cohort.

**Table S10.** Baseline characteristics of non-censored patients between HAIC+Len+ICI and Len groups in the entire cohort.

**Table S11.** Baseline characteristics of censored patients between HAIC+Len+ICI and Len groups in the PSM cohort.

**Table S12.** Baseline characteristics of non-censored patients between HAIC+Len+ICI and Len groups in the PSM cohort.

**Table S13.** Progression patterns in Len and HAIC+Len+ICI groups in entire and PSM cohorts.

**Table S14.** Baseline characteristics of advanced HCC in entire and PSM cohorts.

**Table S15.** The median survival, 6-month, 12-month and 18-month of PFS in three types of advanced HCC in PSM cohort between the two groups.

**Table S16.** The median survival, 1-years, 2-year and 3-year of OS in three types of advanced HCC in PSM cohort between the two groups.

**Table S17.** Most common treatment-related adverse events of patients in two groups in the entire cohort.

**Figure Legends**

**Figure S1**. The standard mean difference (SMD) in the entire, propensity score match (PSM) cohort, inverse probability of treatment weighting (IPTW) cohort, coarsened exact matching (CEM) cohort. it was well balanced after IPTW with all less than 0.1.

**Figure S2**. Stratification analysis of progression-free survival (PFS) in propensity score match (PSM) cohort.

**Figure S3**. Stratification analysis of overall survival (OS) in propensity score match cohort.

**Figure S4**. (A) Landmark analysis of overall survival at 6 months in the entire cohort. (B) Landmark analysis of overall survival at 12 months in the entire cohort.

**Figure S5**. (A) Landmark analysis of overall survival at 6 months in the propensity score match (PSM) cohort. (B) Landmark analysis of overall survival at 12 months in the PSM cohort.

**Figure S6**. A directed acyclic graph represents associations between confounders and treatment modality and survival. Gray circles represent observed confounders, grayish circles represent potential confounders, and gray quadrate represents mediator. Green lines represent directed path, and purple lines represent nondirected paths.

**Figure S7**. Survival curves of post progression survival in entire cohort (A) and propensity score match cohort (B) of three progression patterns.

**Figure S8**. (A) Survival curve of overall survival (OS) in the three types of advanced hepatocellular carcinoma. (B) OS of type I between the Len and HAIC+Len+ICI groups. (C) OS of type II between the Len and HAIC+Len+ICI groups. (D) OS of type III between the Len and HAIC+Len+ICI groups.

**Figure S9**. Stratification analysis of progression-free survival (PFS) in advanced hepatocellular carcinoma.

**Figure S10**. Stratification analysis of overall survival (OS) in advanced hepatocellular carcinoma.

**Table S1.** Baseline characteristics of IPTW cohort and ECM cohort.

| **Characteristics** | **IPTW cohort** | | | **ECM cohort** | | |
| --- | --- | --- | --- | --- | --- | --- |
|  | **Len** | **HAIC+Len+ICI** | ***P* value** | **Len** | **HAIC+Len+ICI** | ***P* value** |
|  | **(n=639)** | **(n=639)** |  | **(n=202)** | **(n=236)** |  |
| **Sex** |  |  | 0.998 |  |  | 0.230 |
| Male | 570 (89.2%) | 570 (89.2%) |  | 183 (90.6%) | 204 (86.4%) |  |
| Female | 69 (10.8%) | 69 (10.8%) |  | 19 (9.4%) | 32 (13.6%) |  |
| **Age, year** |  |  | 0.981 |  |  | 0.267 |
| ≤ 60 | 526 (82.3%) | 527 (82.5%) |  | 164 (81.2%) | 202 (85.6%) |  |
| > 60 | 113 (17.7%) | 112 (17.5%) |  | 38 (18.8%) | 34 (14.4%) |  |
| **Hepatitis** |  |  | 0.910 |  |  | 0.705 |
| No | 79 (12.4%) | 77 (12.1%) |  | 30 (14.9%) | 31 (13.1%) |  |
| Yes | 560 (87.6%) | 562 (87.9%) |  | 172 (85.1%) | 205 (86.9%) |  |
| **Anti-virus** |  |  | 0.895 |  |  | 1.000 |
| No | 154 (24.1%) | 151 (23.6%) |  | 50 (24.8%) | 59 (25.0%) |  |
| Yes | 485 (75.9%) | 488 (76.4%) |  | 152 (75.2%) | 177 (75.0%) |  |
| **Tumor number** |  |  | 0.970 |  |  | 0.856 |
| ≤ 3 | 204 (31.9%) | 203 (31.8%) |  | 54 (26.7%) | 66 (28.0%) |  |
| > 3 | 435 (68.1%) | 436 (68.2%) |  | 148 (73.3%) | 170 (72.0%) |  |
| **Tumor size**, cm |  |  | 0.999 |  |  | 0.848 |
| ≤ 5 | 158 (24.7%) | 159 (24.9%) |  | 47 (23.3%) | 56 (23.7%) |  |
| > 5, ≤ 10 | 256 (40.1%) | 255 (39.9%) |  | 84 (41.6%) | 92 (39.0%) |  |
| > 10 | 225 (35.2%) | 225 (35.2%) |  | 71 (35.1%) | 88 (37.3%) |  |
| **HCC type** |  |  | 0.987 |  |  | 1.000 |
| Recurrent | 177 (27.7%) | 177 (27.7%) |  | 50 (24.8%) | 59 (25.0%) |  |
| Primary | 462 (72.3%) | 462 (72.3%) |  | 152 (75.2%) | 177 (75.0%) |  |
| **Cirrhosis** |  |  | 0.972 |  |  | 1.000 |
| No | 291 (45.5%) | 292 (45.7%) |  | 102 (50.5%) | 120 (50.8%) |  |
| Yes | 348 (54.5%) | 347 (54.3%) |  | 100 (49.5%) | 116 (49.2%) |  |
| **Portal hypertension** |  |  | 0.998 |  |  | 0.959 |
| No | 456 (71.4%) | 456 (71.4%) |  | 151 (74.8%) | 178 (75.4%) |  |
| Yes | 183 (28.6%) | 183 (28.6%) |  | 51 (25.2%) | 58 (24.6%) |  |
| **MVI** |  |  | 0.972 |  |  | 0.760 |
| No | 211 (33.0%) | 211 (33.0%) |  | 69 (34.2%) | 85 (36.0%) |  |
| Yes | 428 (67.0%) | 428 (67.0%) |  | 133 (65.8%) | 151 (64.0%) |  |
| **Metastasis** |  |  | 0.958 |  |  | 1.000 |
| No | 241 (37.7%) | 243 (38.0%) |  | 83 (41.1%) | 96 (40.7%) |  |
| Yes | 398 (62.3%) | 396 (62.0%) |  | 119 (58.9%) | 140 (59.3%) |  |
| **HBV DNA** |  |  | 0.988 |  |  | 1.000 |
| Negative | 458 (71.7%) | 458 (71.7%) |  | 143 (70.8%) | 167 (70.8%) |  |
| Positive | 181 (28.3%) | 181 (28.3%) |  | 59 (29.2%) | 69 (29.2%) |  |
| **AFP**, ng/mL |  |  | 0.979 |  |  | 0.386 |
| ≤ 400 | 291 (45.5%) | 292 (45.7%) |  | 94 (46.5%) | 99 (41.9%) |  |
| > 400 | 348 (54.5%) | 347 (54.3%) |  | 108 (53.5%) | 137 (58.1%) |  |
| **ALT**, U/L |  |  | 0.931 |  |  | 0.290 |
| ≤ 40 | 300 (46.9%) | 298 (46.6%) |  | 89 (44.1%) | 117 (49.6%) |  |
| > 40 | 339 (53.1%) | 341 (53.4%) |  | 113 (55.9%) | 119 (50.4%) |  |
| **AST**, U/L |  |  | 0.997 |  |  | 0.793 |
| ≤ 40 | 203 (31.8%) | 203 (31.8%) |  | 66 (32.7%) | 81 (34.3%) |  |
| > 40 | 436 (68.2%) | 436 (68.2%) |  | 136 (67.3%) | 155 (65.7%) |  |
| **ALBI grade** |  |  | 1.000 |  |  | 0.724 |
| Grade 1 | 224 (35.1%) | 224 (35.1%) |  | 66 (32.7%) | 83 (35.2%) |  |
| Grade 2 | 375 (58.7%) | 375 (58.7%) |  | 133 (65.8%) | 151 (64.0%) |  |
| Grade 3 | 40 (6.3%) | 40 (6.3%) |  | 3 (1.5%) | 2 (0.8%) |  |

Abbreviations: AFP, alpha-fetoprotein; ALBI, albumin-bilirubin; ALT, alanine aminotransferase; AST, aspartate aminotransferase; HBV DNA, hepatitis B virus deoxyribonucleic acid; Len, Lenvatinib; MVI, macrovascular invasion.

**Table S2.** The median survival, 6-month, 12-month and 24-month of PFS between two groups.

| **Study factor** | **Group** | **Median, (95% CI)** | **6-month** | **12-month** | **24-month** |
| --- | --- | --- | --- | --- | --- |
| **Entire cohort** | **Len** | 5.9± 0.2 (5.6-6.2) | 48.0% | 12.6% | 2.0% |
|  | **HAIC+Len+ICI** | 9.2 ± 0.5 (8.3-10.1) | 73.6% | 34.5% | 16.2% |
| **PSM cohort** | **Len** | 5.9± 0.2 (5.6-6.2) | 47.9% | 12.7% | 5.6% |
|  | **HAIC+Len+ICI** | 8.9 ± 0.5 (8.0-9.9) | 73.6% | 34.8% | 15.4% |
| **IPTW cohort** | **Len** | 5.8± 0.2 (5.5-6.2) | 47.7% | 13.1% | 2.2% |
|  | **HAIC+Len+ICI** | 8.9 ± 0.6 (8.2-10.3) | 72.9% | 34.2% | 15.6% |
| **ECM cohort** | **Len** | 6.0± 0.3 (5.7-6.7) | 51.3% | 13.2% | 1.7% |
|  | **HAIC+Len+ICI** | 9.7 ± 0.7 (8.2-11.3) | 72.0% | 34.9% | 11.4% |

**Table S3.** Univariable and multivariable analysis of prognostic factors for PFS in entire cohort.

|  | **HR (95%CI)** | ***P* value** | **HR (95%CI)** | ***P* value** |
| --- | --- | --- | --- | --- |
| **Sex** |  |  |  |  |
| Female vs. male | 1.00 (0.76, 1.30) | 0.975 |  |  |
| **Age, year** |  |  |  |  |
| > 60 vs. ≤ 60 | 0.94 (0.76, 1.18) | 0.608 |  |  |
| **Group** |  |  |  |  |
| HAIC+Len+ICI vs. Len | 0.50 (0.43, 0.60) | <0.001 | 0.47 (0.40-0.56) | <0.001 |
| **Hepatitis** |  |  |  |  |
| Yes vs. no | 1.01 (0.79, 1.30) | 0.929 |  |  |
| **Anti-virus** |  |  |  |  |
| Yes vs. no | 0.86 (0.71, 1.04) | 0.129 |  |  |
| **Tumor size**, cm |  |  |  |  |
| ≤ 5 | Reference |  | 1 |  |
| > 5, ≤ 10 | 1.17 (0.94, 1.44) | 0.152 | 1.35 (1.07-1.69) | 0.010 |
| > 10 | 1.26 (1.02, 1.56) | 0.036 | 1.30 (1.02-1.64) | 0.030 |
| **HCC type** |  |  |  |  |
| Primary vs. recurrent | 1.11 (0.92, 1.33) | 0.266 |  |  |
| **Tumor number** |  |  |  |  |
| > 3 vs. ≤ 3 | 1.40 (1.17, 1.68) | <0.001 | 1.28 (1.06-1.55) | 0.009 |
| **Cirrhosis** |  |  |  |  |
| Yes vs. no | 0.86 (0.73, 1.02) | 0.076 | 0.91 (0.76-1.08) | 0.288 |
| **Portal hypertension** |  |  |  |  |
| Yes vs. no | 0.91 (0.76, 1.09) | 0.317 |  |  |
| **MVI** |  |  |  |  |
| Yes vs. no | 1.16 (0.98, 1.38) | 0.081 | 0.93 (0.75-1.16) | 0.530 |
| **Metastasis** |  |  |  |  |
| Yes vs. no | 1.39 (1.17, 1.65) | <0.001 | 1.49 (1.21-1.84) | <0.001 |
| **HBV DNA** |  |  |  |  |
| Positive vs. negative | 1.08 (0.90, 1.30) | 0.412 |  |  |
| **AFP**, ng/mL |  |  |  |  |
| > 400 vs. ≤ 400 | 1.14 (0.97, 1.34) | 0.123 |  |  |
| **ALT**, U/L |  |  |  |  |
| > 40 vs. ≤ 40 | 1.04 (0.88, 1.23) | 0.620 |  |  |
| **AST**, U/L |  |  |  |  |
| > 40 vs. ≤ 40 | 1.42 (1.19, 1.70) | <0.001 | 1.47 (1.21-1.79) | <0.001 |
| **ALBI grade** |  |  |  |  |
| Grade 1 | Reference |  | 1 |  |
| Grade 1 | 1.18 (0.99, 1.41) | 0.064 | 0.99 (0.82-1.19) | 0.931 |
| Grade 1 | 1.42 (0.99, 2.02) | 0.055 | 0.95 (0.66-1.38) | 0.802 |

Abbreviations: AFP, alpha-fetoprotein; ALBI, albumin-bilirubin; ALT, alanine aminotransferase; AST, aspartate aminotransferase; HBV DNA, hepatitis B virus deoxyribonucleic acid; Len, Lenvatinib; MVI, macrovascular invasion.

**Table S4.** Univariable and multivariable analysis of prognostic factors for PFS in PSM cohort.

|  | **HR (95%CI)** | ***P* value** | **HR (95%CI)** | ***P* value** |
| --- | --- | --- | --- | --- |
| **Sex** |  |  |  |  |
| Female vs. male | 1.09 (0.82, 1.44) | 0.564 |  |  |
| **Age, year** |  |  |  |  |
| > 60 vs. ≤ 60 | 0.94 (0.75, 1.18) | 0.602 |  |  |
| **Group** |  |  |  |  |
| HAIC+Len+ICI vs. Len | 0.51 (0.43, 0.61) | <0.001 | 0.46 (0.38-0.55) | <0.001 |
| **Hepatitis** |  |  |  |  |
| Yes vs. no | 1.00 (0.77, 1.29) | 0.988 |  |  |
| **Anti-virus** |  |  |  |  |
| Yes vs. no | 0.87 (0.71, 1.06) | 0.158 |  |  |
| **Tumor size**, cm |  |  |  |  |
| ≤ 5 | Reference |  |  |  |
| > 5, ≤ 10 | 1.23 (0.98, 1.54) | 0.077 | 1.91 (1.20-3.04) | 0.006 |
| > 10 | 1.28 (1.01, 1.62) | 0.042 | 1.83 (1.10-3.05) | 0.020 |
| **HCC type** |  |  |  |  |
| Primary vs. recurrent | 1.18 (0.97, 1.44) | 0.099 | 1.36 (0.87-2.12) | 0.180 |
| **Tumor number** |  |  |  |  |
| > 3 vs. ≤ 3 | 1.38 (1.13, 1.67) | 0.001 | 1.34 (1.09-1.65) | 0.005 |
| **Cirrhosis** |  |  |  |  |
| Yes vs. no | 0.85 (0.71, 1.01) | 0.069 | 1.07 (0.90-1.29) | 0.441 |
| **Portal hypertension** |  |  |  |  |
| Yes vs. no | 0.92 (0.76, 1.12) | 0.434 |  |  |
| **MVI** |  |  |  |  |
| Yes vs. no | 0.90 (0.75, 1.08) | 0.249 |  |  |
| **Metastasis** |  |  |  |  |
| Yes vs. no | 1.33 (1.11, 1.60) | 0.002 | 1.43 (1.19-1.73) | <0.001 |
| **HBV positive** |  |  |  |  |
| Yes vs. no | 1.14 (0.94, 1.39) | 0.188 |  |  |
| **AFP**, ng/mL |  |  |  |  |
| > 400 vs. ≤ 400 | 1.17 (0.98, 1.39) | 0.087 | 1.03 (0.85-1.24) | 0.787 |
| **ALT**, U/L |  |  |  |  |
| > 40 vs. ≤ 40 | 1.04 (0.87, 1.23) | 0.694 |  |  |
| **AST**, U/L |  |  |  |  |
| > 40 vs. ≤ 40 | 1.36 (1.12, 1.64) | 0.002 | 1.41 (1.14-1.74) | 0.002 |
| **ALBI grade** |  |  |  |  |
| Grade 1 | Reference |  |  |  |
| Grade 2 | 1.12 (0.92, 1.35) | 0.255 |  |  |
| Grade 3 | 1.31 (0.90, 1.90) | 0.156 |  |  |

Abbreviations: AFP, alpha-fetoprotein; ALBI, albumin-bilirubin; ALT, alanine aminotransferase; AST, aspartate aminotransferase; HBV DNA, hepatitis B virus deoxyribonucleic acid; Len, Lenvatinib; MVI, macrovascular invasion.

**Table S5.** The median survival, 1-years, 2-year and 3-year of OS between two groups.

| **Study factor** | **Group** | **Median, (95% CI)** | **1-year** | **2-year** | **3-year** |
| --- | --- | --- | --- | --- | --- |
| **Entire cohort** | Len | 13.3± 0.2 (12.9-13.8) | 61.7% | 15.2% | 7.9% |
|  | HAIC+Len+ICI | 22.0 ± 0.7 (20.7-23.3) | 83.9% | 44.7% | 18.7% |
| **PSM cohort** | Len | 13.3 ± 0.3 (12.8-13.8) | 62.3% | 15.3% | 7.8% |
|  | HAIC+Len+ICI | 22.0 ± 0.8 (20.5-23.5) | 84.6% | 43.9% | 19.2% |
| **IPTW cohort** | Len | 13.4± 0.2 (12.8-13.8) | 47.7% | 13.1% | 2.2% |
|  | HAIC+Len+ICI | 22.0 ± 0.6 (21.0-23.6) | 72.9% | 34.2% | 10.6% |
| **ECM cohort** | Len | 13.6± 0.2 (12.0-14.1) | 51.3% | 13.2% | 1.7% |
|  | HAIC+Len+ICI | 21.9 ± 0.8 (20.1-24.6) | 72.0% | 34.9% | 11.4% |

**Table S6.** Univariable and multivariable analysis of prognostic factors for OS in entire cohort.

|  | **HR (95%CI)** | ***P* value** | **HR (95%CI)** | ***P* value** |
| --- | --- | --- | --- | --- |
| **Sex** |  |  |  |  |
| Female vs. male | 1.10 (0.82, 1.48) | 0.515 |  |  |
| **Age, year** |  |  |  |  |
| > 60 vs. ≤ 60 | 1.01 (0.79, 1.29) | 0.934 |  |  |
| **Group** |  |  |  |  |
| HAIC+Len+ICI vs. Len | 0.38 (0.31, 0.46) | <0.001 | 0.33 (0.27-0.41) | <0.001 |
| **Hepatitis** |  |  |  |  |
| Yes vs. no | 0.92 (0.70, 1.21) | 0.555 |  |  |
| **Anti-virus** |  |  |  |  |
| Yes vs. no | 0.86 (0.69, 1.06) | 0.165 |  |  |
| **Tumor size**, cm |  |  |  |  |
| ≤ 5 | Reference |  |  |  |
| > 5, ≤ 10 | 1.42 (1.11, 1.82) | 0.005 | 2.16 (1.28-3.64) | 0.004 |
| > 10 | 1.71 (1.33, 2.20) | <0.001 | 2.34 (1.33-4.12) | 0.003 |
| **HCC type** |  |  |  |  |
| Primary vs. recurrent | 1.46 (1.18, 1.81) | <0.001 | 1.40 (0.86-2.28) | 0.178 |
| **Tumor number** |  |  |  |  |
| > 3 vs. ≤ 3 | 1.43 (1.17, 1.76) | <0.001 | 1.37 (1.10-1.70) | 0.005 |
| **Cirrhosis** |  |  |  |  |
| Yes vs. no | 0.97 (0.80, 1.16) | 0.718 |  |  |
| **Portal hypertension** |  |  |  |  |
| Yes vs. no | 1.15 (0.94, 1.41) | 0.176 |  |  |
| **MVI** |  |  |  |  |
| Yes vs. no | 1.20 (0.99, 1.46) | 0.065 | 1.13 (0.92-1.39) | 0.233 |
| **Metastasis** |  |  |  |  |
| Yes vs. no | 1.10 (0.91, 1.33) | 0.326 |  |  |
| **HBV DNA** |  |  |  |  |
| Positive vs. negative | 1.44 (1.18, 1.76) | <0.001 | 1.17 (0.95-1.45) | 0.145 |
| **AFP**, ng/mL |  |  |  |  |
| > 400 vs. ≤ 400 | 1.19 (0.99, 1.44) | 0.063 | 1.07 (0.87-1.31) | 0.511 |
| **ALT**, U/L |  |  |  |  |
| > 40 vs. ≤ 40 | 1.21 (1.00, 1.46) | 0.046 | 1.34 (1.07-1.68) | 0.012 |
| **AST**, U/L |  |  |  |  |
| > 40 vs. ≤ 40 | 1.68 (1.37, 2.07) | <0.001 | 1.56 (1.20-2.02) | <0.001 |
| **ALBI grade** |  |  |  |  |
| Grade 1 | Reference |  |  |  |
| Grade 2 | 1.36 (1.11, 1.66) | 0.003 | 1.13 (0.92-1.39) | 0.241 |
| Grade 3 | 2.34 (1.61, 3.40) | <0.001 | 1.61 (1.10-2.36) | 0.014 |

Abbreviations: AFP, alpha-fetoprotein; ALBI, albumin-bilirubin; ALT, alanine aminotransferase; AST, aspartate aminotransferase; HBV DNA, hepatitis B virus deoxyribonucleic acid; Len, Lenvatinib; MVI, macrovascular invasion.

**Table S7.** Univariable and multivariable analysis of prognostic factors for OS in PSM cohort.

|  | **HR (95%CI)** | ***P* value** | **HR (95%CI)** | ***P* value** |
| --- | --- | --- | --- | --- |
| **Sex** |  |  |  |  |
| Female vs. male | 1.13 (0.82, 1.55) | 0.444 |  |  |
| **Age, year** |  |  |  |  |
| > 60 vs. ≤ 60 | 1.03 (0.80, 1.32) | 0.835 |  |  |
| **Group** |  |  |  |  |
| HAIC+Len+ICI vs. Len | 0.34 (0.31-0.46) | <0.001 | 0.34 (0.27-0.42) | <0.001 |
| **Hepatitis** |  |  |  |  |
| Yes vs. no | 0.90 (0.68, 1.20) | 0.480 |  |  |
| **Anti-virus** |  |  |  |  |
| Yes vs. no | 0.87 (0.70, 1.09) | 0.231 |  |  |
| **Tumor size, cm** |  |  |  |  |
| ≤ 5 |  |  |  |  |
| > 5, ≤ 10 | 1.45 (1.11, 1.89) | 0.006 | 2.19 (2.19-3.71) | 0.004 |
| > 10 | 1.65 (1.26, 2.16) | <0.001 | 2.40 (1.35-4.26) | 0.003 |
| **HCC type** |  |  |  |  |
| Primary vs. recurrent | 1.41 (1.12, 1.77) | 0.003 | 0.94 (0.64-1.39) | 0.768 |
| **Tumor number** |  |  |  |  |
| > 3 vs. ≤ 3 | 1.44 (1.15, 1.81) | 0.001 | 1.40 (1.10-1.76) | 0.006 |
| **Cirrhosis** |  |  |  |  |
| Yes vs. no | 0.99 (0.81, 1.20) | 0.886 |  |  |
| **Portal hypertension** |  |  |  |  |
| Yes vs. no | 1.16 (0.94, 1.44) | 0.172 |  |  |
| **MVI** |  |  |  |  |
| Yes vs. no | 1.24 (1.01, 1.53) | 0.046 | 1.25 (1.01-1.55) | 0.046 |
| **Metastasis** |  |  |  |  |
| Yes vs. no | 1.08 (0.88, 1.32) | 0.469 |  |  |
| **HBV DNA** |  |  |  |  |
| positive vs. negative | 1.47 (1.19, 1.82) | <0.001 | 1.23 (0.98-1.55) | 0.073 |
| **AFP, ng/mL** |  |  |  |  |
| > 400 vs. ≤ 400 | 1.27 (1.04, 1.54) | 0.020 | 1.05 (0.85-1.30) | 0.672 |
| **ALT, U/L** |  |  |  |  |
| > 40 vs. ≤ 40 | 1.14 (0.94, 1.39) | 0.184 |  |  |
| **AST, U/L** |  |  |  |  |
| > 40 vs. ≤ 40 | 1.52 (1.22, 1.89) | <0.001 | 1.18 (0.93-1.51) | 0.171 |
| **ALBI grade** |  |  |  |  |
| Grade 1 | Reference |  |  |  |
| Grade 2 | 1.27 (1.02, 1.58) | 0.034 | 1.17 (0.94-1.46) | 0.171 |
| Grade 3 | 2.16 (1.46, 3.18) | <0.001 | 1.65 (1.11-2.46) | 0.013 |

Abbreviations: AFP, alpha-fetoprotein; ALBI, albumin-bilirubin; ALT, alanine aminotransferase; AST, aspartate aminotransferase; HBV DNA, hepatitis B virus deoxyribonucleic acid; Len, Lenvatinib; MVI, macrovascular invasion.

**Table S8.** Results of mediation analysis on the mediation effect of three-month and six-month evaluation in PFS and OS.

|  |  |  | **Total effect (95%CI) *P*** **value** | **Mediation effect (95%CI) *P* value** | | **Direct effect (95%CI) *P* value** |
| --- | --- | --- | --- | --- | --- | --- |
| **Entire Cohort** | PFS | Three-month evaluation | 3.83 (2.92-4.73) *P* <0.0001 | | 2.18 (1.66-2.74) *P* <0.0001 | 1.64 (0.84-2.44) *P* =0.0001 |
|  | PFS | Six-month evaluation | 3.83 (2.92-4.73) *P* <0.0001 | | 2.82 (2.17-3.51) *P* <0.0001 | 1.00 (0.33-1.68) *P* <0.0001 |
|  | OS | Three-month evaluation | 6.09 (4.95-7.24) *P* <0.0001 | | 2.10 (1.51-2.65) *P* <0.0001 | 3.99 (2.89-5.10) *P* <0.0001 |
|  | OS | Six-month evaluation | 6.09 (4.95-7.24) *P* <0.0001 | | 2.79 (2.12-3.51) *P* <0.0001 | 3.30 (2.29-4.31) *P* <0.0001 |
| **PSM Cohort** | PFS | Three-month evaluation | 3.74 (2.80-4.68) *P* <0.0001 | | 2.16 (1.61-2.75) *P* <0.0001 | 1.58 (0.74-2.41) *P* =0.002 |
|  | PFS | Six-month evaluation | 3.74 (2.80-4.68) *P* <0.0001 | | 2.81 (2.10-3.52) *P* <0.0001 | 0.93 (0.24-1.63) *P* =0.009 |
|  | OS | Three-month evaluation | 6.05 (4.84-7.25) *P* <0.0001 | | 2.07 (1.52-2.69) P <0.0001 | 3.97 (2.80-5.14) *P* <0.0001 |
|  | OS | Six-month evaluation | 6.05 (4.84-7.25) *P* <0.0001 | | 2.84 (2.09-3.63) *P* <0.0001 | 2.21 (2.15-4.26) *P* <0.0001 |

**Table S9.** Baseline characteristics of censored patients between HAIC+Len+ICI and Len groups in the entire cohort.

|  | **Len**  **(n=22)** | **HAIC+Len+ICI**  **(n=45)** | ***P* value** |
| --- | --- | --- | --- |
| **Sex** |  |  | 0.119 |
| Male | 17 (77.3%) | 41 (91.1%) |  |
| Female | 5 (22.7%) | 4 (8.9%) |  |
| **Age, year** |  |  | 0.524 |
| ≤ 60 | 19 (86.4%) | 36 (80.0%) |  |
| > 60 | 3 (13.6%) | 9 (20.0%) |  |
| **Hepatitis** |  |  | 0.978 |
| No | 2 (9.1%) | 4 (8.9%) |  |
| Yes | 20 (90.9%) | 41 (91.1%) |  |
| **Anti-virus** |  |  | 0.601 |
| No | 4 (18.2%) | 6 (13.3%) |  |
| Yes | 18 (81.8%) | 39 (86.7%) |  |
| **Tumor size, cm** |  |  | 0.221 |
| ≤ 5 | 8 (36.4%) | 8 (17.8%) |  |
| > 5, ≤ 10 | 9 (40.9%) | 21 (46.6%) |  |
| > 10 | 5 (22.7%) | 16 (35.6%) |  |
| **HCC type** |  |  | 0.055 |
| Recurrent | 8 (36.4%) | 7 (15.6%) |  |
| Primary | 14 (63.6%) | 38 (84.4%) |  |
| **Tumor number** |  |  | 0.044 |
| ≤ 3 | 6 (27.3%) | 24 (53.3%) |  |
| > 3 | 16 (72.7%) | 21 (46.7%) |  |
| **Cirrhosis** |  |  | 0.667 |
| No | 8 (36.4%) | 14 (31.1%) |  |
| Yes | 14 (63.6%) | 31 (68.9%) |  |
| **Portal hypertension** |  |  | 0.308 |
| No | 16 (72.7%) | 27 (60.0%) |  |
| Yes | 6 (27.3%) | 18 (40.0%) |  |
| **MVI** |  |  | 0.007 |
| No | 7 (31.8%) | 3 (6.7%) |  |
| Yes | 15 (68.2%) | 42 (93.3%) |  |
| **Metastasis** |  |  | 0.045 |
| No | 9 (40.9%) | 30 (66.7%) |  |
| Yes | 13 (59.1%) | 15 (33.3%) |  |
| **HBV DNA** |  |  | 0.373 |
| Negative | 17 (77.3%) | 30 (66.7%) |  |
| Positive | 5 (22.7%) | 15 (33.3%) |  |
| **AFP, ng/mL** |  |  | 0.437 |
| ≤ 400 | 12 (54.5%) | 20 (44.4%) |  |
| > 400 | 10 (45.5%) | 25 (55.6%) |  |
| **ALT, U/L** |  |  | 0.261 |
| ≤ 40 | 12 (54.5%) | 18 (40.0%) |  |
| > 40 | 10 (45.5%) | 27 (60.0%) |  |
| **AST, U/L** |  |  | 0.438 |
| ≤ 40 | 11 (50.0%) | 18 (40.0%) |  |
| > 40 | 11 (50.0%) | 27 (60.0%) |  |
| **ALBI grade** |  |  | 0.752 |
| Grade 1 | 8 (36.4%) | 17 (37.8%) |  |
| Grade 2 | 12 (54.5%) | 26 (57.8%) |  |
| Grade 3 | 2 (9.1%) | 2 (4.4%) |  |
| **Tumor size**, cm | 7.6 ± 4.3 | 9.260 ± 4.471 | 0.160 |
| **AFP**, ng/mL | 9800.8 ± 20480.1 | 49618.5 ± 183729.3 | 0.328 |
| **ALB**, g/L | 37.9 ± 3.6 | 38.220 ± 3.913 | 0.745 |
| **ALT**, U/L | 49.3 ± 38.1 | 66.6 ± 75.4 | 0.325 |
| **AST**, U/L | 60.8 ± 42.7 | 97.1 ± 125.5 | 0.204 |
| **Cre**, mg/dL | 80.1 ± 40.2 | 67.5 ± 11.5 | 0.057 |
| **TBIL**, µmol/L | 29.3 ± 57.8 | 17.3 ± 10.3 | 0.176 |
| **HGB**, g/L | 130.9 ± 18.3 | 138.7 ± 16.1 | 0.083 |
| **NEU,** x10^9^ | 4.6 ± 2.3 | 3.7 ± 1.9 | 0.122 |
| **PLT,** x10^9^ | 203.8 ± 162.7 | 181.91 ± 99.5 | 0.502 |

Abbreviations: AFP, alpha-fetoprotein; ALBI, albumin-bilirubin; ALT, alanine aminotransferase; AST, aspartate aminotransferase; HBV DNA, hepatitis B virus deoxyribonucleic acid; Len, Lenvatinib; MVI, macrovascular invasion.

**Table S10.** Baseline characteristics of non-censored patients between HAIC+Len+ICI and Len groups in the entire cohort.

|  | **Len**  **(n=268)** | **HAIC+Len+ICI**  **(n=304)** | ***P* value** |
| --- | --- | --- | --- |
| **Sex** |  |  | 0.484 |
| Male | 242 (90.3%) | 269 (88.5%) |  |
| Female | 26 (9.7%) | 35 (11.5%) |  |
| **Age**, year |  |  | 0.650 |
| ≤ 60 | 221 (82.5%) | 255 (83.9%) |  |
| > 60 | 47 (17.5%) | 49 (16.1%) |  |
| **Hepatitis** |  |  | 0.343 |
| No | 37 (13.8%) | 34 (11.2%) |  |
| Yes | 231 (86.2%) | 270 (88.8%) |  |
| **Anti-virus** |  |  | 0.391 |
| No | 70 (26.1%) | 70 (23.0%) |  |
| Yes | 198 (73.9%) | 234 (77.0%) |  |
| **Tumor size**, cm |  |  | 0.957 |
| ≤ 5 | 68 (25.4%) | 74 (24.3%) |  |
| > 5, ≤ 10 | 106 (39.5%) | 121 (39.8%) |  |
| > 10 | 94 (35.1%) | 109 (35.9%) |  |
| **HCC type** |  |  | 0.789 |
| Recurrent | 74 (27.6%) | 87 (28.618%) |  |
| Primary | 194 (72.4%) | 217 (71.382%) |  |
| **Tumor number** |  |  | 0.577 |
| ≤ 3 | 78 (29.1%) | 95 (31.3%) |  |
| > 3 | 190 (70.9%) | 209 (68.7%) |  |
| **Cirrhosis** |  |  | 0.450 |
| No | 122 (45.5%) | 148 (48.7%) |  |
| Yes | 146 (54.5%) | 156 (51.3%) |  |
| **Portal hypertension** |  |  | 0.194 |
| No | 188 (70.1%) | 228 (75.0%) |  |
| Yes | 80 (29.9%) | 76 (25.0%) |  |
| **MVI** |  |  | 0.272 |
| No | 87 (32.5%) | 112 (36.8%) |  |
| Yes | 181 (67.5%) | 192 (63.2%) |  |
| **Metastasis** |  |  | 0.796 |
| No | 98 (36.6%) | 108 (35.5%) |  |
| Yes | 170 (63.4%) | 196 (64.5%) |  |
| **HBV DNA** |  |  | 0.926 |
| Negative | 194 (72.4%) | 219 (72.039%) |  |
| Positive | 74 (27.6%) | 85 (27.961%) |  |
| **AFP**, ng/mL |  |  | 0.048 |
| ≤ 400 | 135 (50.4%) | 128 (42.105%) |  |
| > 400 | 133 (49.6%) | 176 (57.895%) |  |
| **ALT**, U/L |  |  | 0.216 |
| ≤ 40 | 121 (45.149%) | 153 (50.329%) |  |
| > 40 | 147 (54.851%) | 151 (49.671%) |  |
| **AST**, U/L |  |  | 0.426 |
| ≤ 40 | 79 (29.478%) | 99 (32.566%) |  |
| > 40 | 189 (70.522%) | 205 (67.434%) |  |
| **ALBI grade** |  |  | <0.001 |
| Grade 1 | 78 (29.1%) | 121 (39.8%) |  |
| Grade 2 | 164 (61.2%) | 173 (56.9%) |  |
| Grade 3 | 26 (9.7%) | 10 (3.3%) |  |
| **Tumor size**, cm | 8.7 ± 4.4 | 8.733 ± 4.482 | 0.914 |
| **AFP**, ng/mL | 27888.8 ± 86974.8 | 29236.9 ± 93749.5 | 0.861 |
| **ALB**, g/L | 37.2 ± 4.4 | 37.9 ± 4.6 | 0.060 |
| **ALT**, U/L | 68.9 ± 121.1 | 63.8 ± 87.4 | 0.557 |
| **AST**, U/L | 111.4 ± 309.1 | 89.8 ± 113.4 | 0.259 |
| **Cre**, mg/dL | 70.7 ± 21.9 | 69.3 ± 24.1 | 0.472 |
| **TBIL**, µmol/L | 21.2 ± 20.1 | 18.6 ± 20.9 | 0.132 |
| **HGB**, g/L | 134.9 ± 71.3 | 133.7 ± 25.6 | 0.788 |
| **NEU,** x10^9^ | 4.8 ± 2.6 | 4.7 ± 3.1 | 0.771 |
| **PLT,** x10^9^ | 207.1 ± 153.1 | 191.2 ± 96.2 | 0.137 |

Abbreviations: AFP, alpha-fetoprotein; ALBI, albumin-bilirubin; ALT, alanine aminotransferase; AST, aspartate aminotransferase; HBV DNA, hepatitis B virus deoxyribonucleic acid; Len, Lenvatinib; MVI, macrovascular invasion.

**Table S11.** Baseline characteristics of censored patients between HAIC+Len+ICI and Len groups in the PSM cohort.

|  | **Len**  **(n=22)** | **HAIC+Len+ICI**  **(n=35)** | ***P*-value** |
| --- | --- | --- | --- |
| **Sex** |  |  | 0.017 |
| Male | 17 (77.3%) | 34 (97.1%) |  |
| Female | 5 (22.7%) | 1 (2.9%) |  |
| **Age**, year |  |  | 0.539 |
| ≤ 60 | 19 (86.4%) | 28 (80.0%) |  |
| > 60 | 3 (13.6%) | 7 (20.0%) |  |
| **Hepatitis** |  |  | 0.780 |
| No | 2 (9.1%) | 4 (11.4%) |  |
| Yes | 20 (90.9%) | 31 (88.6%) |  |
| **Anti-virus** |  |  | 0.920 |
| No | 4 (18.2%) | 6 (17.1%) |  |
| Yes | 18 (81.8%) | 29 (82.9%) |  |
| **Tumor size**, cm |  |  | 0.224 |
| ≤ 5 | 8 (36.4%) | 6 (17.1%) |  |
| > 5, ≤ 10 | 9 (40.9%) | 16 (45.7%) |  |
| > 10 | 5 (22.7%) | 13 (37.2%) |  |
| **HCC type** |  |  | 0.269 |
| Recurrent | 8 (36.4%) | 8 (22.857%) |  |
| Primary | 14 (63.6%) | 27 (77.143%) |  |
| **Tumor number** |  |  | 0.072 |
| ≤ 3 | 6 (27.3%) | 18 (51.4%) |  |
| > 3 | 16 (72.7%) | 17 (48.6%) |  |
| **Cirrhosis** |  |  | 0.700 |
| No | 8 (36.4%) | 11 (31.4%) |  |
| Yes | 14 (63.6%) | 24 (68.6%) |  |
| **Portal hypertension** |  |  | 0.327 |
| No | 16 (72.7%) | 21 (60.0%) |  |
| Yes | 6 (27.3%) | 14 (40.0%) |  |
| **MVI** |  |  | 0.025 |
| No | 7 (31.8%) | 3 (8.6%) |  |
| Yes | 15 (68.2%) | 32 (91.4%) |  |
| **Metastasis** |  |  | 0.160 |
| No | 9 (40.9%) | 21 (60.0%) |  |
| Yes | 13 (59.1%) | 14 (40.0%) |  |
| **HBV DNA** |  |  | 0.626 |
| Negative | 17 (77.3%) | 25 (71.4%) |  |
| Positive | 5 (22.7%) | 10 (28.6%) |  |
| **AFP**, ng/mL |  |  | 0.516 |
| ≤ 400 | 12 (54.5%) | 16 (45.7%) |  |
| > 400 | 10 (45.5%) | 19 (54.3%) |  |
| **ALT**, U/L |  |  | 0.283 |
| ≤ 40 | 12 (54.5%) | 14 (40.0%) |  |
| > 40 | 10 (45.5%) | 21 (60.0%) |  |
| **AST**, U/L |  |  | 0.339 |
| ≤ 40 | 11 (50.0%) | 13 (37.1%) |  |
| > 40 | 11 (50.0%) | 22 (62.9%) |  |
| **ALBI grade** |  |  | 0.686 |
| Grade 1 | 8 (36.3%) | 10 (28.6%) |  |
| Grade 2 | 12 (54.5%) | 23 (65.7%) |  |
| Grade 3 | 2 (9.1%) | 2 (5.7%) |  |
| **Tumor size**, cm | 7.623 ± 4.340 | 9.483 ± 4.816 | 0.146 |
| **AFP**, ng/mL | 9800.8 ± 20480.1 | 60146.087 ± 208046.709 | 0.276 |
| **ALB**, g/L | 37.9 ± 3.6 | 37.5 ± 3.5 | 0.725 |
| **ALT**, U/L | 49.3 ± 38.1 | 68.9 ± 80.9 | 0.302 |
| **AST**, U/L | 60.8 ± 42.7 | 103.7 ± 135.5 | 0.166 |
| **Cre**, mg/dL | 80.1 ± 40.2 | 68.5 ± 10.8 | 0.113 |
| **TBIL**, µmol/L | 29.4 ± 57.8 | 17.4 ± 11.0 | 0.238 |
| **HGB**, g/L | 130.9 ± 18.4 | 137.9 ± 15.5 | 0.132 |
| **NEU,** x10^9^ | 4.67 ± 2.3 | 3.5 ± 1.8 | 0.070 |
| **PLT,** x10^9^ | 203.8 ± 162.8 | 185.6 ± 97.8 | 0.602 |

Abbreviations: AFP, alpha-fetoprotein; ALBI, albumin-bilirubin; ALT, alanine aminotransferase; AST, aspartate aminotransferase; HBV DNA, hepatitis B virus deoxyribonucleic acid; Len, Lenvatinib; MVI, macrovascular invasion.

**Table S12.** Baseline characteristics of non-censored patients between HAIC+Len+ICI and Len groups in the PSM cohort.

|  | **Len**  **(n=258)** | **HAIC+Len+ICI**  **(n=245)** | ***P* value** |
| --- | --- | --- | --- |
| **Sex** |  |  | 0.625 |
| Male | 232 (89.9%) | 217 (88.6%) |  |
| Female | 26 (10.1%) | 28 (11.4%) |  |
| **Age, year** |  |  | 0.935 |
| ≤ 60 | 212 (82.2%) | 202 (82.4%) |  |
| > 60 | 46 (17.8%) | 43 (17.6%) |  |
| **Hepatitis** |  |  | 0.489 |
| No | 37 (14.3%) | 30 (12.2%) |  |
| Yes | 221 (85.7%) | 215 (87.8%) |  |
| **Anti-virus** |  |  | 0.499 |
| No | 70 (27.1%) | 60 (24.5%) |  |
| Yes | 188 (72.9%) | 185 (75.5%) |  |
| **Tumor size**, cm |  |  | 0.564 |
| ≤ 5 | 63 (24.4%) | 51 (20.8%) |  |
| > 5, ≤ 10 | 103 (39.9%) | 107 (43.7%) |  |
| > 10 | 92 (35.7%) | 87 (35.5%) |  |
| **HCC type** |  |  | 0.515 |
| Recurrent | 73 (28.3%) | 63 (25.7%) |  |
| Primary | 185 (71.7%) | 182 (74.3%) |  |
| **Tumor number** |  |  | 0.470 |
| ≤ 3 | 77 (29.8%) | 66 (26.9%) |  |
| > 3 | 181 (70.2%) | 179 (73.1%) |  |
| **Cirrhosis** |  |  | 0.920 |
| No | 121 (46.9%) | 116 (47.3%) |  |
| Yes | 137 (53.1%) | 129 (52.7%) |  |
| **Portal hypertension** |  |  | 0.663 |
| No | 184 (71.3%) | 179 (73.1%) |  |
| Yes | 74 (28.7%) | 66 (26.9%) |  |
| **MVI** |  |  | 0.370 |
| No | 87 (33.7%) | 92 (37.6%) |  |
| Yes | 171 (66.3%) | 153 (62.4%) |  |
| **Metastasis** |  |  | 0.377 |
| No | 95 (36.8%) | 81 (33.1%) |  |
| Yes | 163 (63.2%) | 164 (66.9%) |  |
| **HBV DNA** |  |  | 0.893 |
| Negative | 185 (71.7%) | 177 (72.2%) |  |
| Positive | 73 (28.3%) | 68 (27.8%) |  |
| **AFP**, ng/mL |  |  | 0.738 |
| ≤ 400 | 126 (48.8%) | 116 (47.3%) |  |
| > 400 | 132 (51.2%) | 129 (52.7%) |  |
| **ALT, U/L** |  |  | 0.467 |
| ≤ 40 | 118 (45.7%) | 120 (49.0%) |  |
| > 40 | 140 (54.3%) | 125 (51.0%) |  |
| **AST**, U/L |  |  | 0.629 |
| ≤ 40 | 77 (29.8%) | 78 (31.8%) |  |
| > 40 | 181 (70.2%) | 167 (68.2%) |  |
| **ALBI grade** |  |  | 0.062 |
| Grade 1 | 78 (30.2%) | 82 (33.5%) |  |
| Grade 2 | 156 (60.5%) | 153 (62.4%) |  |
| Grade 3 | 24 (9.3%) | 10 (4.1%) |  |
| **Tumor size**, cm | 8.801 ± 4.381 | 8.933 ± 4.422 | 0.737 |
| **AFP**, ng/mL | 28593.9 ± 88415.7 | 28861.7 ± 102101.5 | 0.975 |
| **ALB**, g/L | 37.3 ± 4.3 | 37.4 ± 4.6 | 0.723 |
| **ALT**, U/L | 65.3 ± 101.3 | 66.5 ± 93.2 | 0.894 |
| **AST**, U/L | 111.4 ± 314.6 | 90.3 ± 117.8 | 0.329 |
| **Cre**, mg/dL | 70.7 ± 22.2 | 69.7 ± 25.9 | 0.639 |
| **TBIL**, µmol/L | 21.2 ± 20.4 | 19.6 ± 22.8 | 0.437 |
| **HGB**, g/L | 135.4 ± 72.5 | 132.1 ± 26.5 | 0.508 |
| **NEU,** x10^9^ | 4.8 ± 2.6 | 4.9 ± 3.2 | 0.613 |
| **PLT,** x10^9^ | 207.6 ± 153.6 | 195.7 ± 100.2 | 0.310 |

Abbreviations: AFP, alpha-fetoprotein; ALBI, albumin-bilirubin; ALT, alanine aminotransferase; AST, aspartate aminotransferase; HBV DNA, hepatitis B virus deoxyribonucleic acid; Len, Lenvatinib; MVI, macrovascular invasion.

**Table S13.** Progression patterns in Len and HAIC+Len+ICI groups in entire and PSM cohorts.

| **Progression patterns** | **Entire cohort** | | | **PSM cohort** | | |
| --- | --- | --- | --- | --- | --- | --- |
|  | **Len**  **(n=264)** | **HAIC+Len+ICI**  **(n=302)** | ***P* value** | **Len (n=255)** | **HAIC+Len+ICI**  **(n=245)** | ***P* value** |
| **Type 1** | 109 (41.3%) | 149 (49.3%) | 0.019 | 105 (41.2%) | 117 (47.8%) | 0.028 |
| **Type 2** | 90 (34.1%) | 106 (35.1%) |  | 86 (33.7%) | 90 (36.7%) |  |
| **Type 3** | 65 (24.6%) | 47 (15.6%) |  | 64 (25.1%) | 38 (15.5%) |  |

Progression patterns were classified into three types. Type 1 was defined as progression on liver-only. Type 2 was defined as progression on non-liver organs (such as bone, lymph nodule, paranephros). Type 3 was defined as liver associated with other non-liver organs.

**Table S14.** Baseline characteristics of advanced HCC in entire and PSM cohorts.

| **Characteristics** | **Entire cohort** | | | **PSM cohort** | | |
| --- | --- | --- | --- | --- | --- | --- |
|  | **Len Group** | **HAIC+Len+PD-1 Group** | ***P* value** | **Len Group** | **HAIC+Len+PD-1 Group** | ***P* value** |
|  | **(n=290)** | **(n=349)** |  | **(n=280)** | **(n=280)** |  |
| **Advanced HCC type** |  |  | 0.704 |  |  | 0.985 |
| MVI only | 107 (36.9%) | 137 (39.3%) |  | 104 (37.1%) | 102 (36.4%) |  |
| Metastasis only | 94 (32.4%) | 115 (33.0%) |  | 94 (33.6%) | 95 (33.9%) |  |
| MVI+Metastasis | 89 (30.7%) | 97 (27.8%) |  | 82 (29.3%) | 83 (29.6%) |  |
| **HVTT** |  |  | 0.181 |  |  | 0.366 |
| No | 246 (84.8%) | 282 (80.8%) |  | 237 (84.6%) | 229 (81.8%) |  |
| Yes | 44 (15.2%) | 67 (19.2%) |  | 43 (15.4%) | 51 (18.2%) |  |
| **Metastasis** |  |  | 0.494 |  |  | 0.861 |
| No | 107 (36.9%) | 138 (39.5%) |  | 104 (37.1%) | 102 (36.4%) |  |
| Yes | 183 (63.1%) | 211 (60.5%) |  | 176 (62.9%) | 178 (63.6%) |  |
| **Lung** |  |  | 0.048 |  |  | 0.036 |
| No | 189 (65.2%) | 194 (55.6%) |  | 181 (64.6%) | 151 (53.9%) |  |
| Unilateral | 24 (8.3%) | 37 (10.6%) |  | 24 (8.6%) | 32 (11.4%) |  |
| Bilateral | 77 (26.6%) | 118 (33.8%) |  | 75 (26.8%) | 97 (34.6%) |  |
| **Bone** |  |  | 0.087 |  |  | 0.026 |
| No | 253 (87.2%) | 319 (91.4%) |  | 243 (86.8%) | 259 (92.5%) |  |
| Yes | 37 (12.8%) | 30 (8.6%) |  | 37 (13.2%) | 21 (7.5%) |  |
| **Lymph node** |  |  | 0.144 |  |  | 0.289 |
| No | 208 (71.7%) | 268 (76.8%) |  | 202 (72.1%) | 213 (76.1%) |  |
| Yes | 82 (28.3%) | 81 (23.2%) |  | 78 (27.9%) | 67 (23.9%) |  |
| **PVTT** |  |  | 0.406 |  |  | 0.980 |
| No | 98 (33.8%) | 119 (34.1%) |  | 98 (35.0%) | 97 (34.6%) |  |
| I | 28 (9.7%) | 41 (11.7%) |  | 28 (10.0%) | 31 (11.1%) |  |
| II | 75 (25.9%) | 101 (28.9%) |  | 72 (25.7%) | 72 (25.7%) |  |
| III | 89 (30.7%) | 88 (25.2%) |  | 82 (29.3%) | 80 (28.6%) |  |

Abbreviations: MVI, macrovascular invasion; PVTT, portal vein tumor thrombus; HVTT, hepatic vein tumor thrombus.

**Table S15.** The median survival, 6-month, 12-month and 18-month of PFS in three types of advanced HCC in PSM cohort between the two groups.

|  |  | **Median PFS (95%CI)** | **6-mon** | **12-month** | **18-month** |
| --- | --- | --- | --- | --- | --- |
| **Total** | Len | 5.9 ± 0.2 (5.6-6.2) | 47.9% | 12.7% | 5.6% |
|  | HAIC+Len+ICI | 8.9 ± 0.5 (7.9-9.9) | 73.6% | 34.8% | 15.4% |
| **MVI only** | Len | 6.2 ± 0.4 (5.4-6.9) | 55.8% | 12.9% | 2.6% |
|  | HAIC+Len+ICI | 11.2 ± 0.7 (9.9-12.6) | 86.3% | 44.7% | 22.1% |
| **Metastasis only** | Len | 6.1 ± 0.4 (5.3-6.9) | 51.8% | 19.1% | 10.4% |
|  | HAIC+Len+ICI | 7.7 ± 0.8 (6.1-9.3) | 64.2% | 30.5% | 8.3% |
| **MVI+Metastasis** | Len | 5.2 ± 0.2 (4.8-5.6) | 33.5% | 4.3% | 2.1% |
|  | HAIC+Len+ICI | 8.7 ± 1.0 (6.7-10.6) | 68.7% | 27.4% | 15.6% |

**Table S16.** The median survival, 1-years, 2-year and 3-year of OS in three types of advanced HCC in PSM cohort between the two groups.

|  |  | **Median OS (95%CI)** | **1-year OS** | **2-year OS** | **3-year OS** |
| --- | --- | --- | --- | --- | --- |
| **Total** | Len | 13.3 ± 0.3 (12.8-13.8) | 62.3% | 15.3% | 7.8% |
|  | HAIC+Len+ICI | 22.0 ± 0.8 (20.5-23.5) | 84.6% | 43.9% | 19.2% |
| **MVI only** | Len | 12.9 ± 0.5 (12.0-13.9) | 62.3% | 14.5% | 7.3% |
|  | HAIC+Len+ICI | 24.1 ± 2.4 (19.4-28.9) | 91.2% | 50.2% | 19.3% |
| **Metastasis only** | Len | 14.3 ± 0.4 (13.5-15.2) | 71.6% | 24.9% | 12.7% |
|  | HAIC+Len+ICI | 22.0 ± 1.0 (20.3-24.1) | 81.1% | 42.7% | 21.5% |
| **MVI+Metastasis** | Len | 12.3 ± 0.9 (10.5-14.1) | 51.1% | 4.2% | 2.1% |
|  | HAIC+Len+ICI | 20.8 ± 1.6 (17.6-23.9) | 80.7% | 37.9% | 10.0% |

**Table S17.** Most common treatment-related adverse events of patients in two groups in the entire cohort.

| **Adverse events** | **Len group**  (n=290) | **HAIC+Len+ICI group**  (n=349) |
| --- | --- | --- |
| **Neutropenia**  Grade 1-2  Grade 3-4 | 18 (6.2)  0 (0.0) | 102 (29.2)  14 (4.0) |
| **Thrombocytopenia**  Grade 1-2  Grade 3-4 | 9 (3.1)  0 (0.0) | 59 (16.9)  12 (3.4) |
| **Nausea**  Grade 1-2  Grade 3-4 | 21 (11.1)  0 (0.0) | 221 (72.0)  10 (5.9) |
| **Vomiting**  Grade 1-2  Grade 3-4 | 19 (6.6)  0 (0.0) | 165 (63.3)  7 (2.0) |
| **Hypertension**  Grade 1-2  Grade 3-4 | 47 (16.2)  7 (2.4) | 53 (15.2)  6 (1.7) |
| **Fatigue**  Grade 1-2  Grade 3-4 | 62 (21.4)  0 (0.0) | 165 (47.3)  9 (2.6) |
| **Rash or desquamation**  Grade 1-2  Grade 3-4 | 29 (10.0)  0 (0.0) | 41 (11.7)  0 (0.0) |
| **Diarrhea**  Grade 1-2  Grade 3-4 | 43 (14.8)  2 (0.7) | 29 (8.3)  3 (0.9) |
| **Decreased appetite**  Grade 1-2  Grade 3-4 | 48 (16.6)  2 (0.7) | 178 (51.0)  7 (2.0) |
| **Elevated ALT**  Grade 1-2  Grade 3-4 | 37 (12.8)  0 (2.6) | 121 (34.7)  15 (4.3) |
| **Elevated AST**  Grade 1-2  Grade 3-4 | 35 (12.1)  3 (1.0) | 93 (61.9)  16 (8.5) |
| **Hyperbilirubinemia**  Grade 1-2  Grade 3-4 | 14 (4.8)  0 (0.0) | 102 (26.6)  10 (2.7) |
| **Proteinuria**  Grade 1-2  Grade 3-4 | 43 (14.8)  5 (1.7) | 54 (15.5)  5 (1.4) |
| **Hypothyroidism**  Grade 1-2  Grade 3-4 | 28 (9.7)  3 (1.0) | 33 (9.5)  5 (1.4) |
| **Weight decreased**  Grade 1-2  Grade 3-4 | 35 (12.1)  2 (0.7) | 72 (20.6)  7 (2.0) |
| **Sensory neuropathy**  Grade 1-2  Grade 3-4 | 0 (0.0)  0 (0.0) | 33 (9.5)  0 (0.0) |

Abbreviations: ALT, alanine aminotransferase; AST, aspartate aminotransferase; Len, Lenvatinib; PD-1, programmed cell death protein-1.


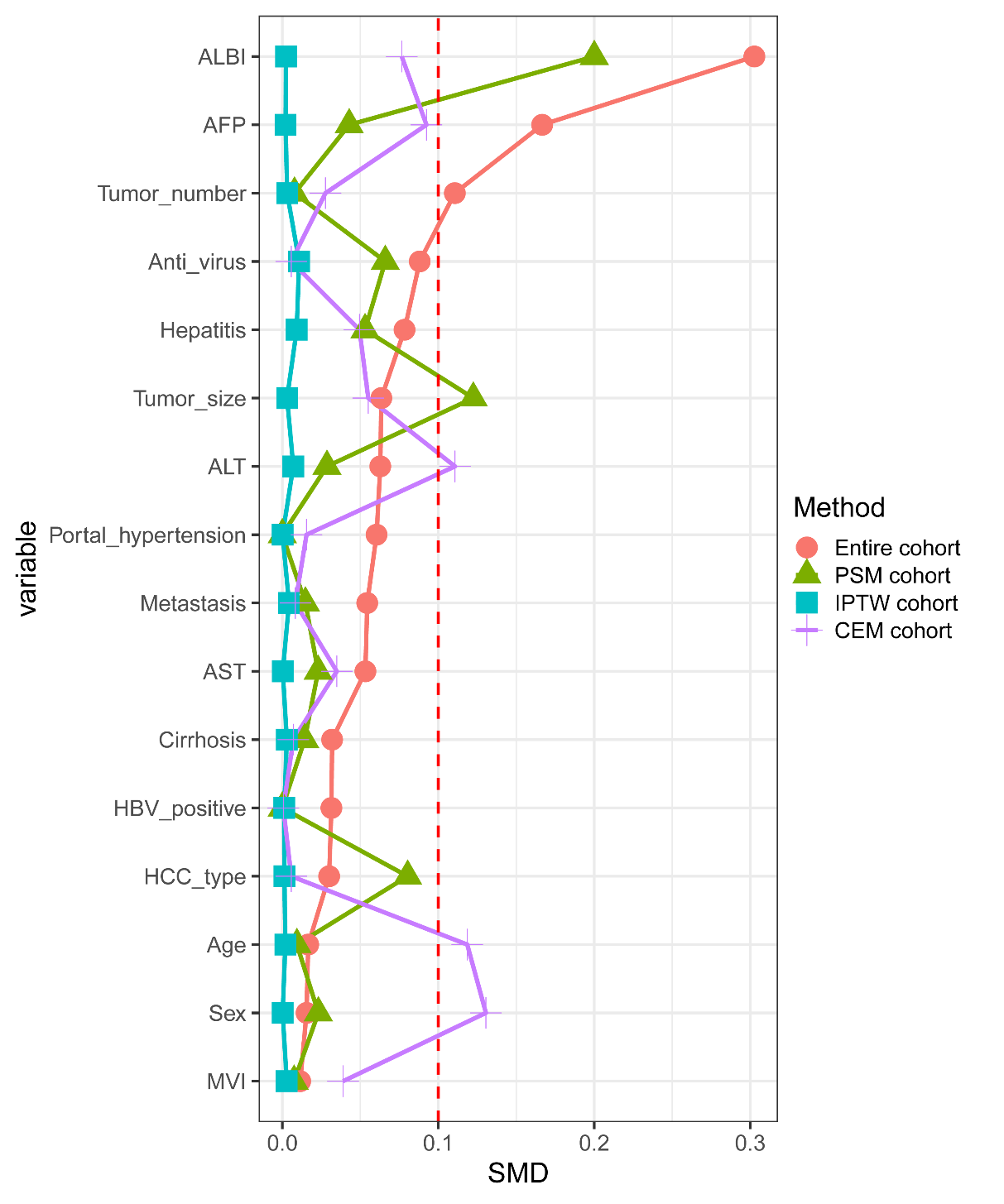


**Figure S1**. The standard mean difference (SMD) in the entire, propensity score match (PSM) cohort, inverse probability of treatment weighting (IPTW) cohort, coarsened exact matching (CEM) cohort. it was well balanced after IPTW with all less than 0.1.


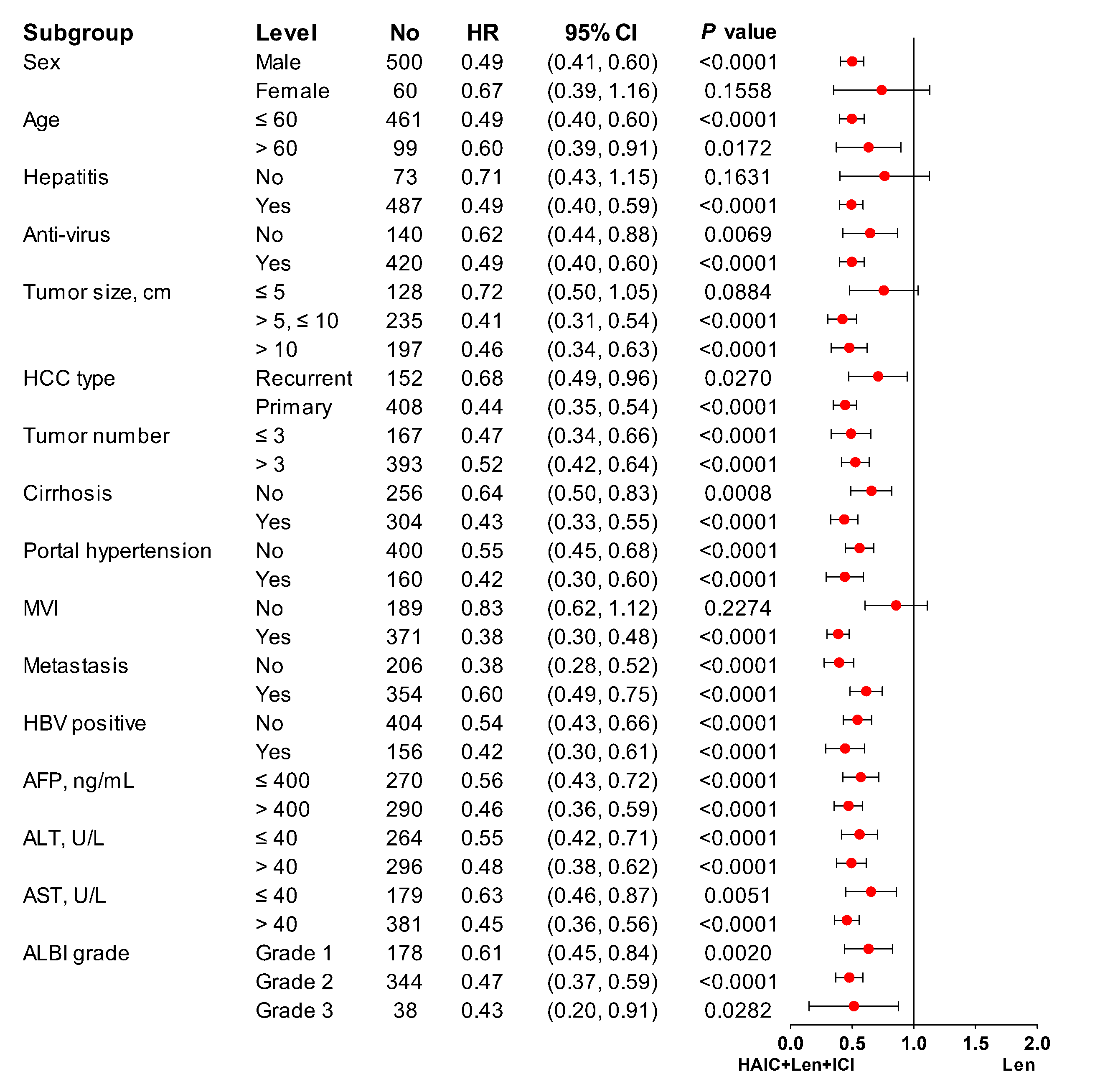


**Figure S2**. Stratification analysis of progression-free survival (PFS) in propensity score match (PSM) cohort. CI, confidence interval; HCC, hepatocellular carcinoma; MVI, macrovascular invasion. HBV, hepatitis B virus; HR, hazard ratio; Len, Lenvatinib; AFP, alpha-fetoprotein; ALT, alanine aminotransferase; AST, aspartate aminotransferase; ALBI, albumin-bilirubin; Len, lenvatinib, HAIC+Len+ICI, hepatic arterial infusion chemotherapy (HAIC) combined with Len and immune checkpoint inhibitor (ICI).


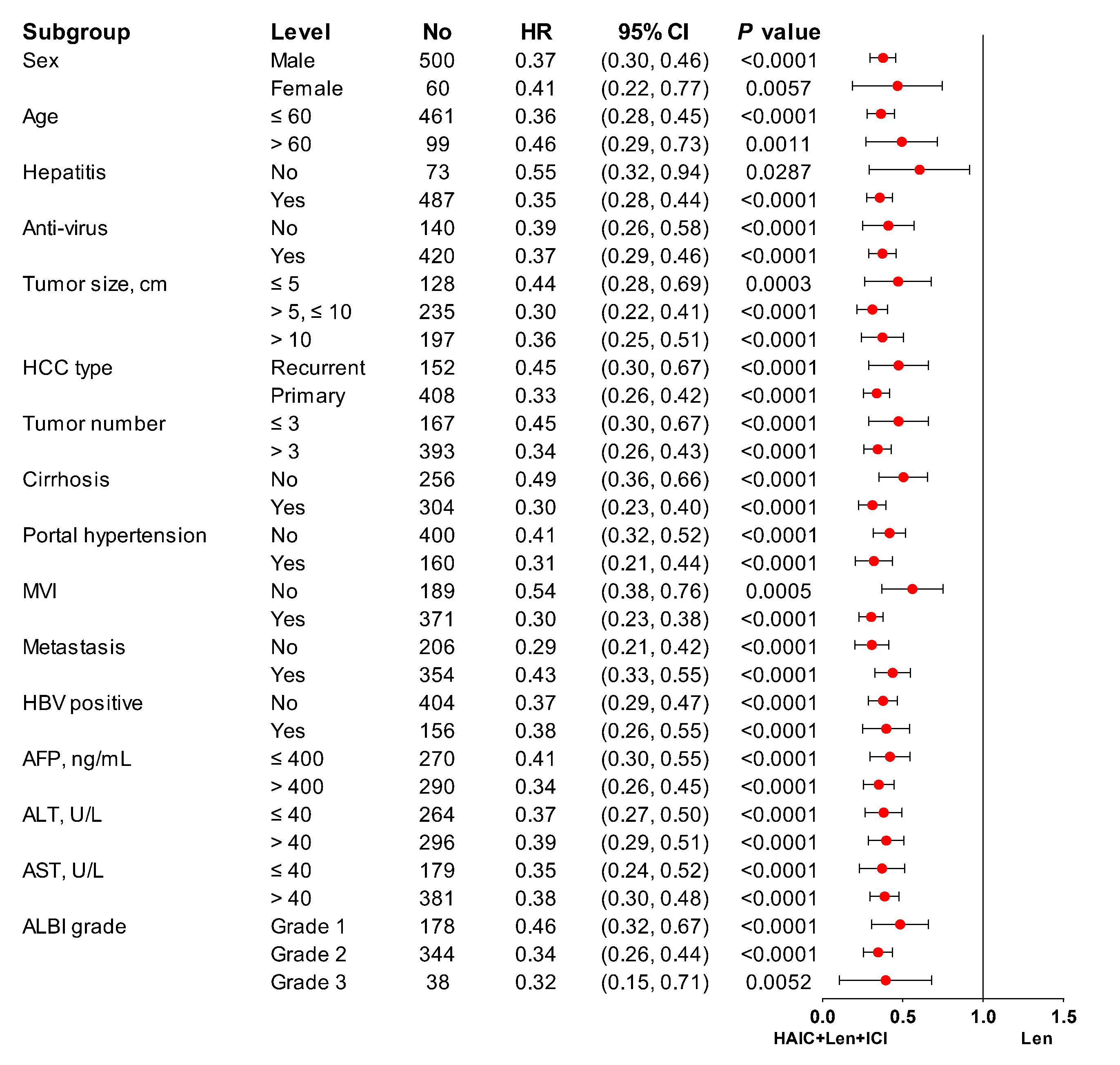


**Figure S3**. Stratification analysis of overall survival (OS) in propensity score match cohort. CI, confidence interval; HCC, hepatocellular carcinoma; MVI, macrovascular invasion. HBV, hepatitis B virus; HR, hazard ratio; Len, Lenvatinib; AFP, alpha-fetoprotein; ALT, alanine aminotransferase; AST, aspartate aminotransferase; ALBI, albumin-bilirubin; Len, lenvatinib, HAIC+Len+ICI, hepatic arterial infusion chemotherapy (HAIC) combined with Len and immune checkpoint inhibitor (ICI).


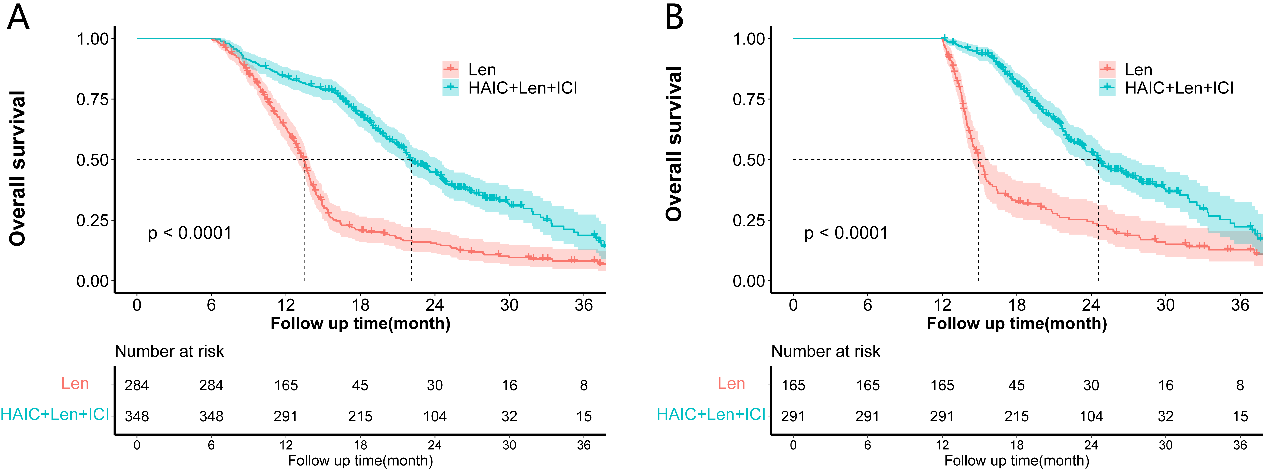


**Figure S4**. (A) Landmark analysis of overall survival at 6 months in the entire cohort. (B) Landmark analysis of overall survival at 12 months in the entire cohort.


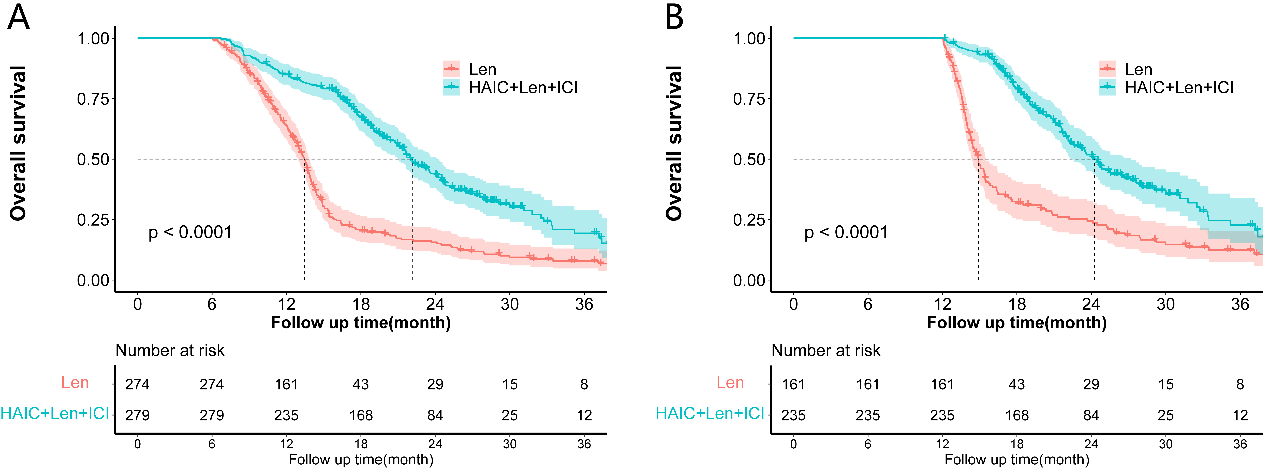


**Figure S5**. (A) Landmark analysis of overall survival at 6 months in the propensity score match (PSM) cohort. (B) Landmark analysis of overall survival at 12 months in the PSM cohort.


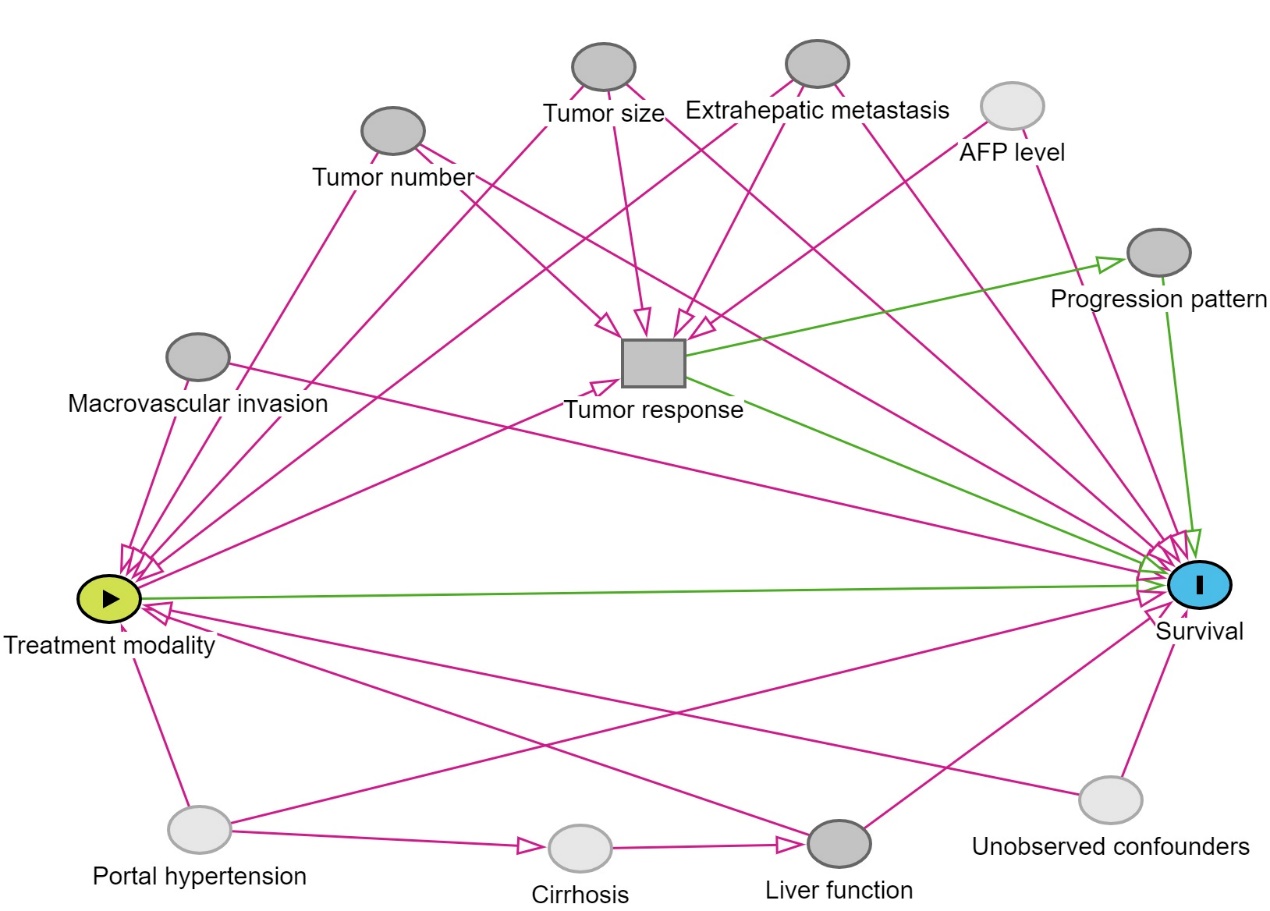


**Figure S6**. A directed acyclic graph represents associations between confounders and treatment modality and survival. Gray circles represent observed confounders, grayish circles represent potential confounders, and gray quadrate represents mediator. Green lines represent directed path, and purple lines represent nondirected paths.


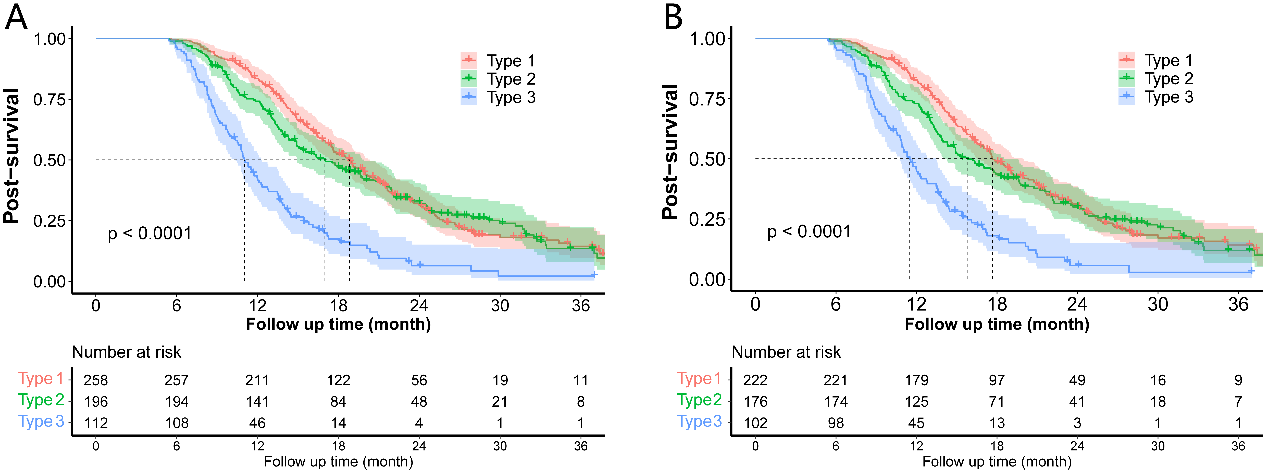


**Figure S7**. Survival curves of post progression survival in entire cohort (A) and propensity score match cohort (B) of three progression patterns. Type 1, progression in the liver only; type 2, progression to non-liver organs (such as bone, lymph nodes, and kidneys); and type 3, liver progression associated with other non-liver organs.


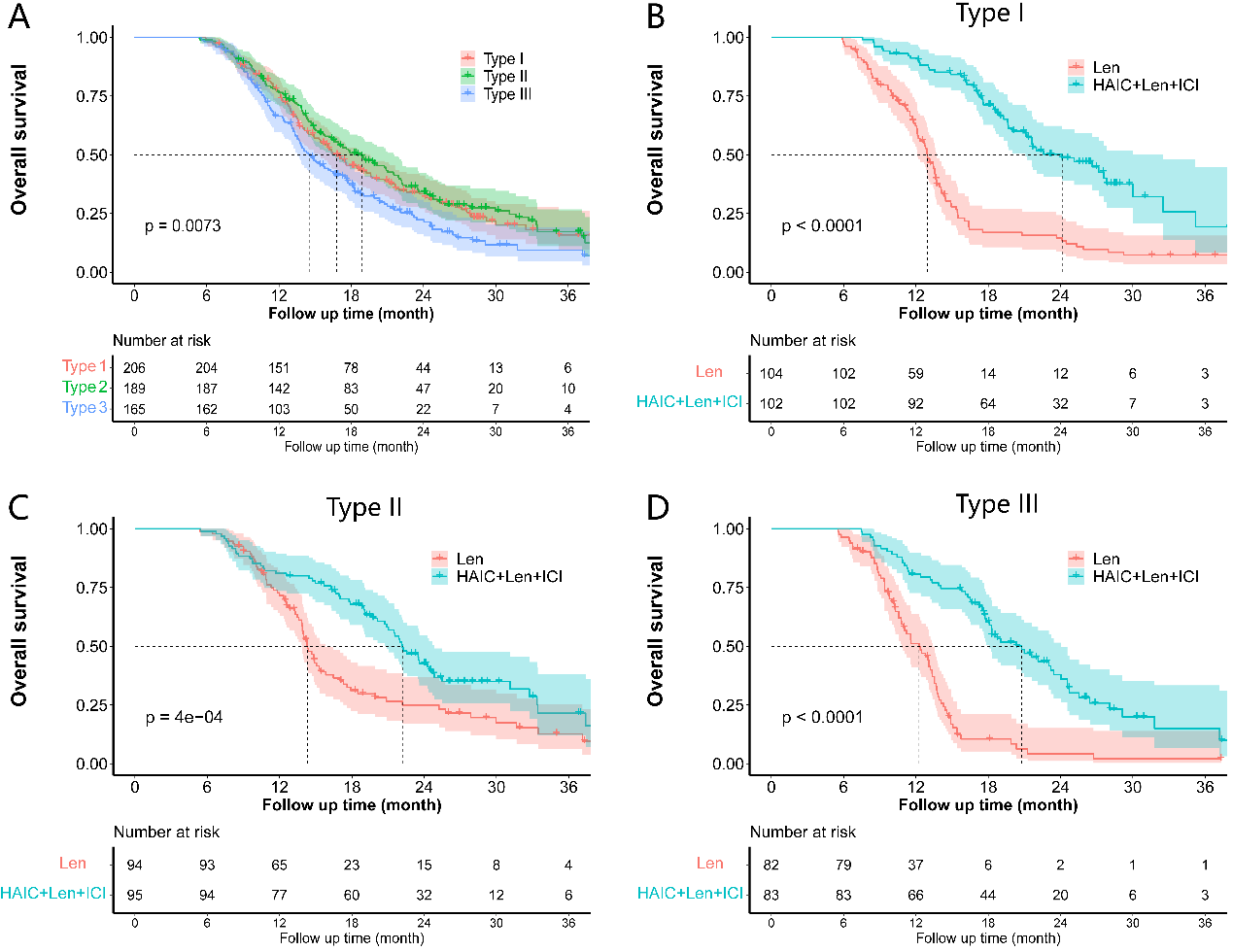


**Figure S8**. (A) Survival curve of overall survival (OS) in the three types of advanced hepatocellular carcinoma. (B) OS of type I between the Len and HAIC+Len+ICI groups. (C) OS of type II between the Len and HAIC+Len+ICI groups. (D) OS of type III between the Len and HAIC+Len+ICI groups. Type I was characterized by localized advanced HCC with macrovascular invasion (MVI) only; type II was defined as HCC with extrahepatic metastases only (including lungs, bones, and lymph nodes); and type III was defined as HCC with MVI and extrahepatic metastasis synchronously.


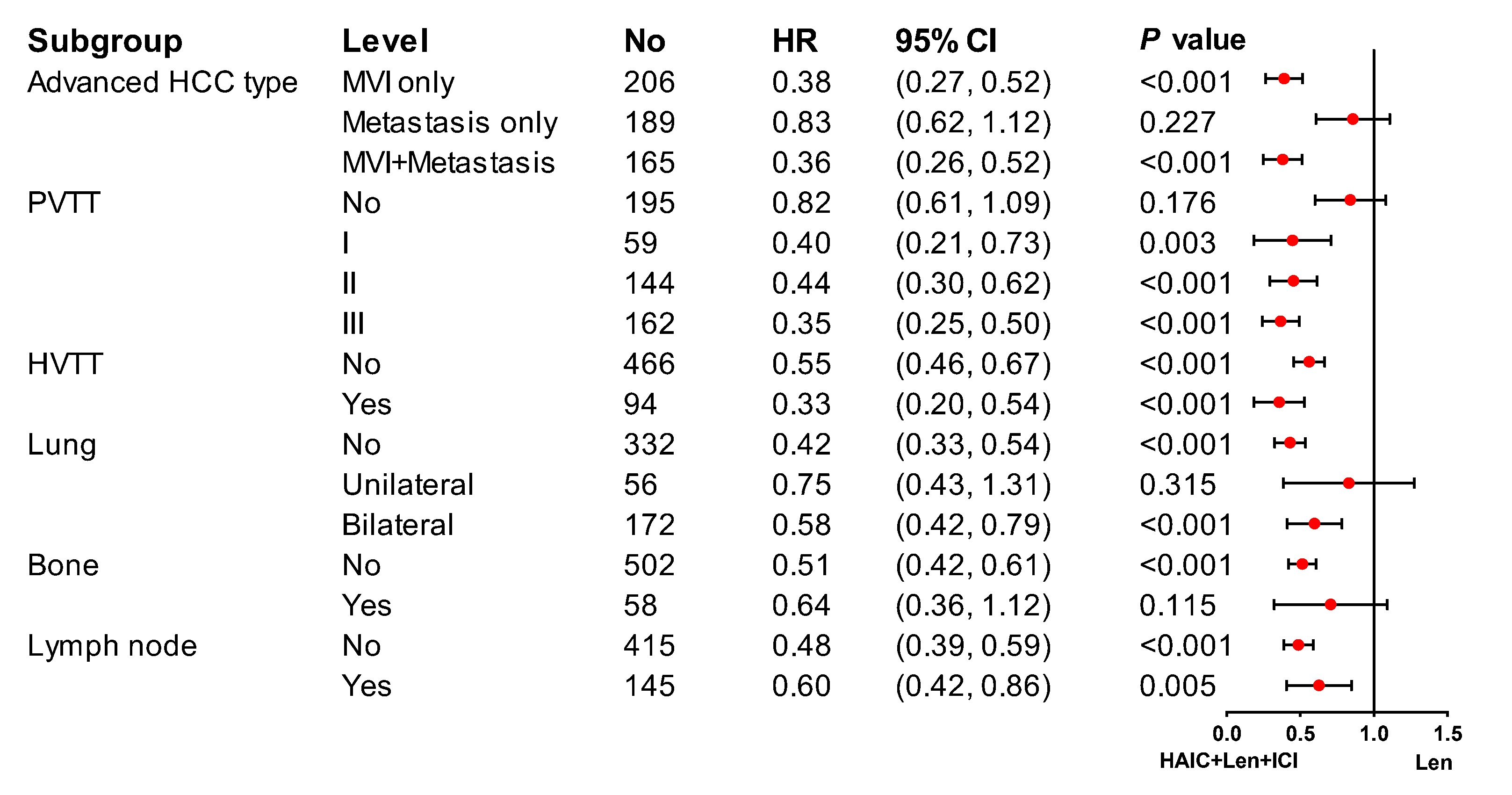
**Figure S9**. Stratification analysis of progression-free survival (PFS) in advanced hepatocellular carcinoma. HCC, hepatocellular carcinoma; MVI, macrovascular invasion. PVTT, portal vein tumor thrombus. HVTT, hepatic vein tumor thrombus. Len, lenvatinib; HAIC+Len+ICI, hepatic arterial infusion chemotherapy (HAIC) combined with Len and immune checkpoint inhibitor (ICI).


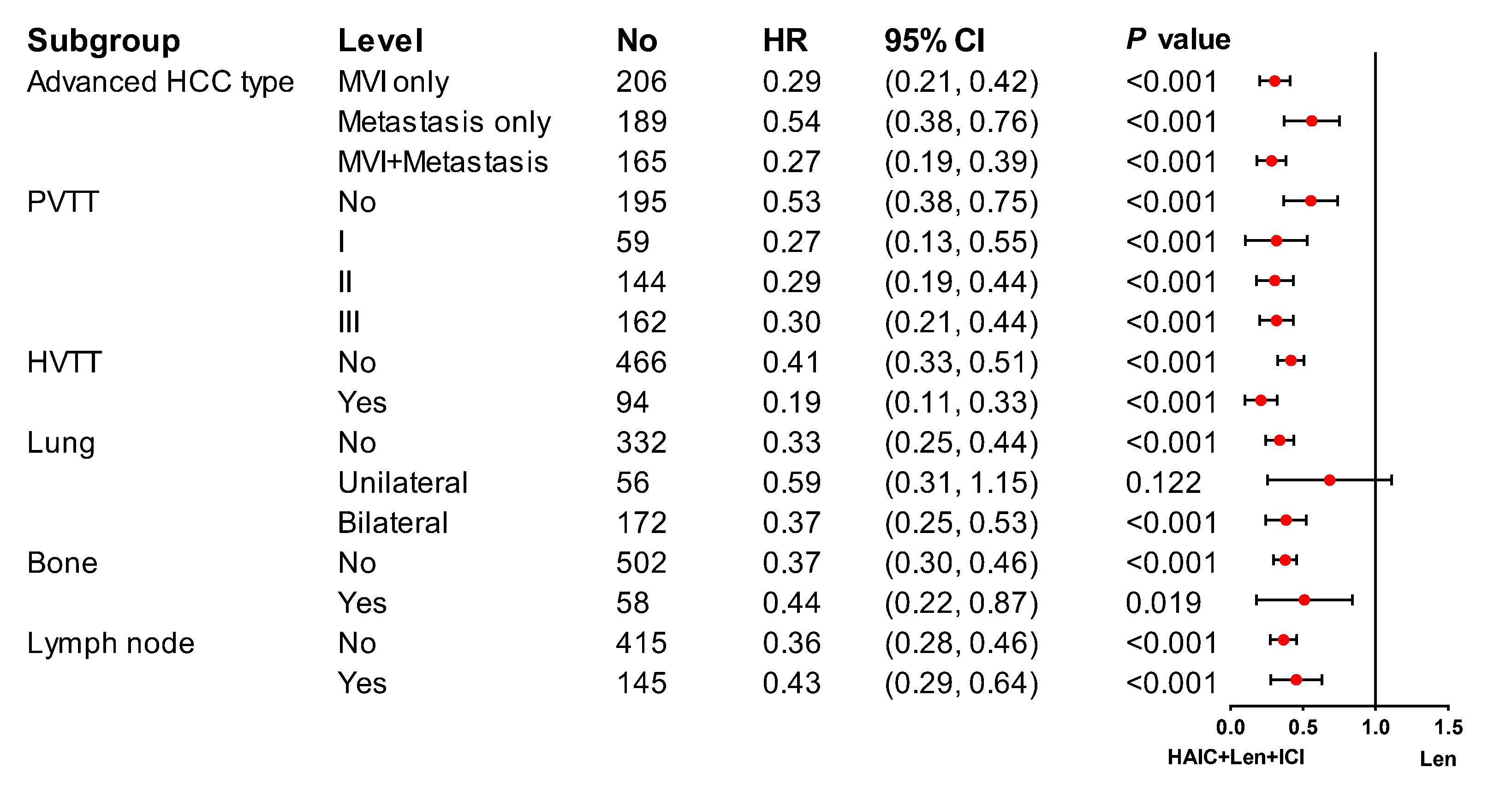


**Figure S10**. Stratification analysis of overall survival (OS) in advanced hepatocellular carcinoma. CI, confidence interval; HR, hazard ratio; HCC, hepatocellular carcinoma; MVI, macrovascular invasion. PVTT, portal vein tumor thrombus. HVTT, hepatic vein tumor thrombus. Len, lenvatinib; HAIC+Len+ICI, hepatic arterial infusion chemotherapy (HAIC) combined with Len and immune checkpoint inhibitor (ICI).
